# Boosting CO_2_ Hydrogenation Activity by Carbon Coating over a Nickel Catalyst

**DOI:** 10.1021/acsomega.6c08194

**Published:** 2026-10-14

**Authors:** Rafael L Oliveira, Karolina A. Ledwa, Olga Chernyayeva, Krzysztof Matus

**Affiliations:** † Institute of Low Temperature and Structure Research of the Polish Academy of Sciences, Wroclaw 50-422, Poland; ‡ 215275 Institute of Physical Chemistry of the Polish Academy of Sciences, Warsaw 01-224, Poland; § Materials Research Laboratory, 49569 Silesian University of Technology, Gliwice 44-100, Poland

## Abstract

Earth-abundant metal-based materials play a crucial role in catalyzing the production of sustainable chemicals and fuels, offering a cost-effective and scalable alternative to precious metals. Among earth-abundant metals, nickel-based catalysts show the biggest potential for industrial applications. Herein, a series of nickel-based catalysts was synthesized by thermal decomposition of Ni complexes over an alumina support under argon, thereby forming a thin carbon layer around the monodispersed nickel particles. By selecting a ligand, the particle size could be tuned (from 10 to 5 nm), and the C-layer structure could be modified by introducing dopants such as N. The solids obtained were used as catalysts for CO_2_ hydrogenation. The material in which Ni particles with a covering C-layer overperformed the “naked” Ni particles at low temperatures. This may be related to the smaller Ni particles obtained from complex decomposition and a synergistic effect between these carbon layers and the Ni particles.

## Graphical abstract

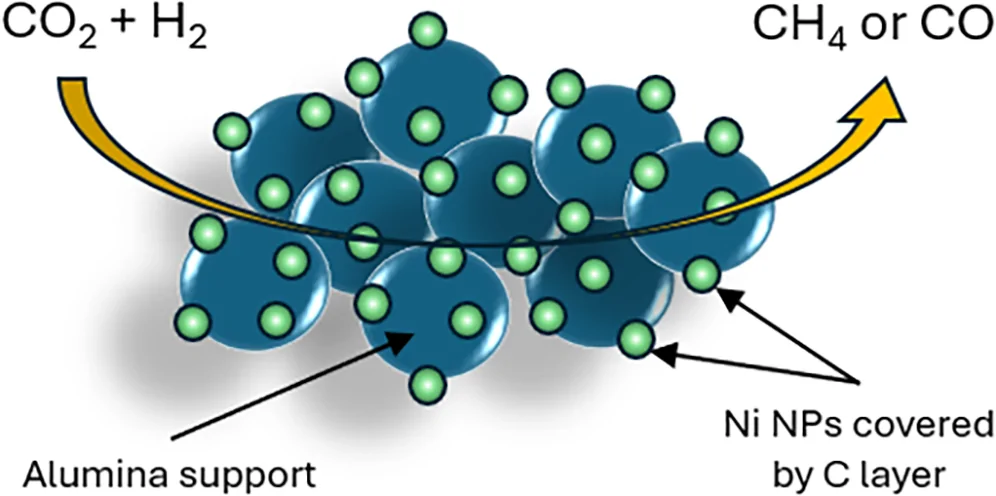

## Introduction

Carbon dioxide hydrogenation offers a promising pathway to convert a potent greenhouse gas into valuable fuels and chemicals, thereby advancing a circular carbon economy. This reaction, typically catalyzed by metal-based heterogeneous catalysts, involves the reductive transformation of thermodynamically stable CO_2_ with hydrogen. The primary products are carbon monoxide, methane (via the Sabatier reaction), methanol, or higher hydrocarbons (via a modified Fischer–Tropsch synthesis).^1–3^

Noble metals such as ruthenium (Ru), palladium (Pd), platinum (Pt), and rhodium (Rh) play a distinctive role due to their exceptional catalytic properties. These metals, often deployed as nanoparticles on advanced supports, exhibit superior activity at relatively low temperatures and demonstrate a remarkable ability to dissociate H_2_, a critical step in activating the inert CO_2_ molecule.^4–10^ However, their high cost limits their application in industrial-scale processes; thus, nonprecious metals are often the focus of scalable CO_2_ hydrogenation.

Metals such as nickel, cobalt, iron, and copper have been extensively studied for CO_2_ hydrogenation.^11–19^ The major scientific challenges lie in designing highly active, selective, and stable catalysts that can operate efficiently at lower temperatures and pressures while mitigating deactivation, thereby improving the overall energy efficiency and economic viability of this crucial carbon capture and utilization strategy.

Among non-noble metals, nickel has the highest potential for industrial applications because of its high activity and relatively low cost. Nickel’s catalytic properties can be tuned by its electronic properties, geometric structures, and particle size.^20,21^ These properties can be adjusted by varying the support-metal interaction by selecting the support on which the Ni particles are immobilized. Regarding Ni particle size, there is ongoing debate and conflicting results regarding its influence on CO_2_ hydrogenation. Some researchers suggested that particles with a size of 2–3 nm on silica^20^ or with an average size of 7–8 nm on carbonaceous material^22^ exhibited the highest catalytic activity; other researchers, however, found little influence of metal particles on catalytic activity.^23–25^

A drawback of nickel catalysts for CO_2_ hydrogenation to produce CO and methane is their relatively high operating temperatures (>400 °C) and pressures (>10 bar). As a consequence, Ni-based catalysts are highly susceptible to deactivation via particle growth, in which nanoparticles agglomerate and lose active surface area at the elevated temperatures required for the reaction.^26–28^ This intrinsic limitation has driven significant research into alloying with promoters, stabilizing catalysts on advanced supports, or developing alternative catalyst systems to overcome these challenges.

Strategies to synthesize more stable catalysts have also been explored, including sophisticated metal precursors such as metal–organic frameworks, covalent organic frameworks, porphyrins, metal complexes, functionalized supports, and metal coverings.^29–41^ These methods typically yield a narrow particle-size distribution and strong metal–support interaction, which limit metal-species mobility and prevent particle growth. Ye et al. reported the deposition of a high-Ni-loading catalyst (using a Ni complex as the metal precursor) on different supports (SiO_2_, ZrO_2_, and Al_2_O_3_). They reported that Ni on zirconia support exhibits the highest catalytic activity among the studied supports and outperforms some noble-metal-based catalysts. They attributed this performance to the enhanced local electron density at the Ni particles arising from strong metal–support interactions.^42^

In this contribution, a series of catalysts was prepared by depositing different Ni complexes onto the surface of commercial AluC-805, a hydrophobic alumina-based material. Subsequent thermal decomposition of the complexes in an argon atmosphere resulted in the formation of monodisperse nickel nanoparticles encapsulated within a thin carbon layer. This synthetic approach enables precise control over the catalyst structure: the choice of ligand governs the size of the resulting Ni particle, while the composition of the carbon layer can be tuned by incorporating heteroatoms such as nitrogen or phosphorus. The catalytic performance of the obtained materials was evaluated in CO_2_ hydrogenation. This system provides a well-defined platform for investigating structure-performance relationships, particularly the effects of nickel particle size and the presence of a carbon overlayer on catalytic activity, product selectivity, and long-term stability.

## Results and Discussion

Alu C-805 was selected as the support due to its hydrophobic character, resulting from surface functionalization with organosilane groups. This property is particularly advantageous in the present synthesis, as several ligands used for Ni complexation are more soluble in organic solvents. Compatibility between the hydrophobic support and these solvents ensures efficient dispersion of Alu C-805, promoting a more uniform distribution of Ni complexes across the Al_2_O_3_ surface.

Figure 1(a) shows a synthetic scheme of a C-covered Ni-supported catalyst. In summary, Ni reacts with the ligand to form a complex, which is then further impregnated into alumina, dried, pyrolyzed under argon, and reduced at 550 °C under a pure hydrogen flow. Table 1 lists the ligands used and the resulting materials.

**Figure 1:**
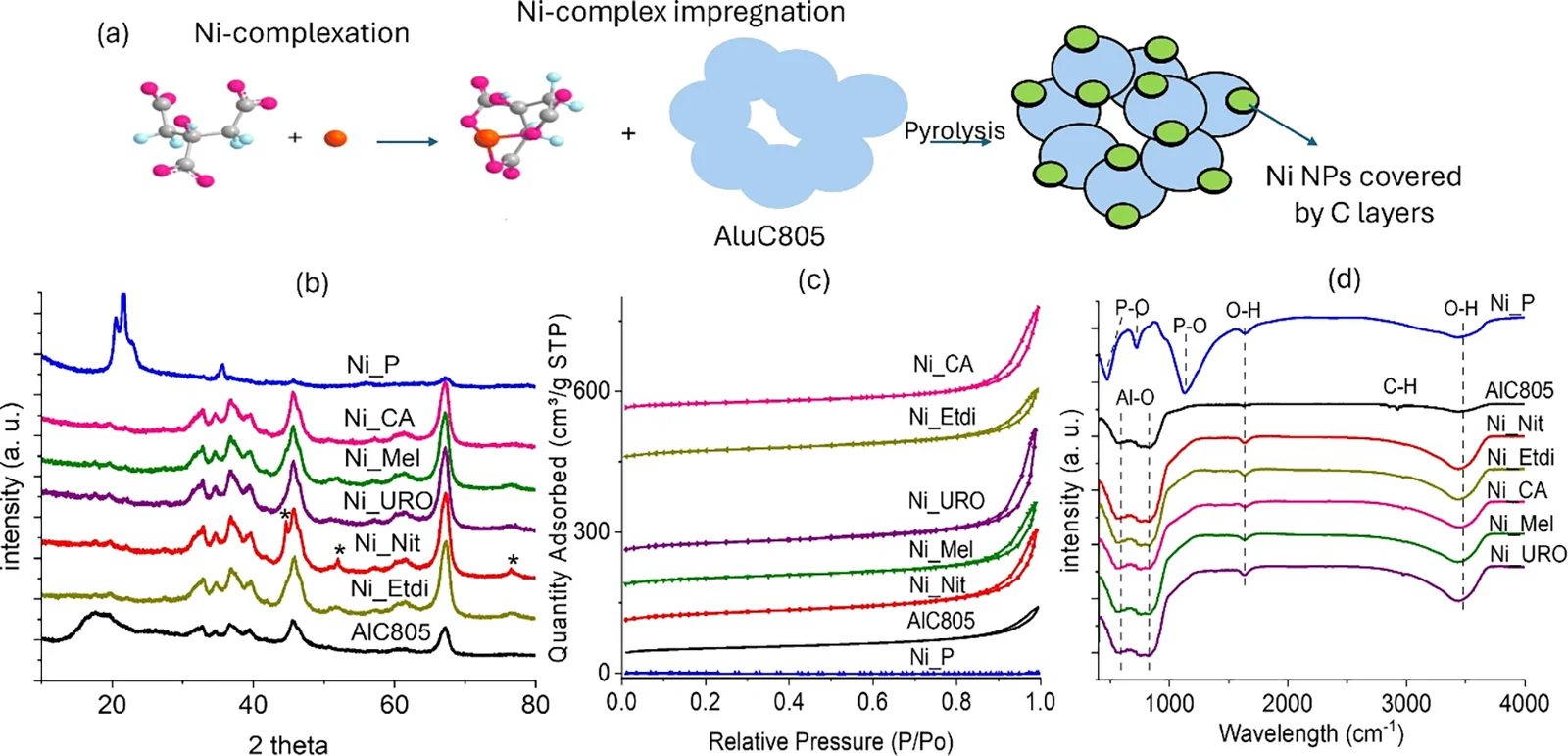
(a) Scheme of synthesis of Ni-based catalysts; (b) XRD pattern (* Ni reflection); (c) N_2_ physisorption isotherms. The isotherms of AlC805, N-Nit, Ni_Mel, Ni_URO, Ni_Etdi, and Ni_CA were offset by 30, 100, 180, 250, 450, and 550 cm^3^/g, respectively; (d) FTIR spectra.

**Table 1:** Nomenclature of Synthesized Materials with Respective Ligand Used, Their Textural Properties, and Ni-Size

Material	Ligand	Surface area (m^2^/g)	Pore volume(cm^3^/g)	Particle size^a^ (nm)	Ni loading (wt.%)^b^
Alu C 805	-	80.6	0.17	-	-
Ni_Nit	-	97.8	0.31	10.1 ± 2.2	5.1
Ni_Etdi	ethylene diamine	86.6	0.23	8.7 ± 2.2	4.1
Ni_URO	urotropine	94.6	0.41	6.3 ± 1.4	4.4
Ni_Mel	melamine	82.5	0.28	6.4 ± 1.3	4.6
Ni_P	phytic acid	1	0	7.7 ± 2.3	3.9
Ni_CA	citric acid	86.2	0.35	4.8 ± 1.3	4.1

a from HAADF-STEM images.

b from atomic absorption spectroscopy (AAS) analysis.

Figure 1 (b) shows the X-ray Diffraction (XRD) pattern of the obtained catalysts and the pristine support. The pristine support shows six major peaks at 2θ values of 32.7°, 36.7°, 39.5°, 45.6°, 61.5°, and 67.2°, corresponding to the reflections (200), (311), (222), (400), (511), and (440) characteristic of γ-alumina.^43^ After the nickel nanoparticle deposition and their reduction, the peaks of alumina are still visible as expected, and two or three very broad and weak peaks related to nickel were observed, located at about 44.5°, 52.1°, and 76.41°, associated with the reflection (111), (220), and (200) planes corresponding to a face-centered cubic structure (PDF 125687). The Ni_Nit sample, in which no complex formation occurred, exhibited sharper, more intense diffraction peaks, indicating larger metal particles dispersed on the support. The alkyl group in the functionalized alumina cannot coordinate the nickel species, suggesting that during pyrolysis they formed a carbon layer on the alumina surface.

When phytic acid was used as a ligand, the alumina support was significantly modified, resulting in the formation of aluminum phosphate, as reported in the literature.^44^ Phytic acid can react with alumina via its phosphate groups, forming Al–O–P bonds. The XRD patterns reveal the disappearance of the characteristic alumina reflections and the appearance of new peaks at 20.6°, 21.7°, and 35.7°, consistent with the orthophosphate phase of AlPO_4_.^45^

Figure 1(c) shows the N_2_ physisorption isotherms of the synthesized catalysts measured at −196.15 °C. The aluminum phosphate exhibits a flat isotherm without hysteresis, indicating that it is nonporous. The alumina-based materials exhibit type IV behavior with an H1 hysteresis loop over the range 0.88–0.99 (relative pressure). This loop is typical of fumed alumina, which consists of relatively dense spheres, with the porous structure comprising the empty spaces between particles. Table 1 shows the surface area and pore volume values, indicating that the materials fall within the same range. Figure S1 shows an exemplary Transmission electron microscopy (TEM) image of the pristine support (Alu C-805) to corroborate the morphology described.

Figure 1(d) displays the Fourier transform infrared spectroscopy (FTIR) spectra of the synthesized materials; all samples (except Ni_P) exhibited two intense bands in the low-wavelength range at about 564 and 790 cm^–1^, which are associated with the stretching vibration of Al–O, typical of Al_2_O_3_. The bands at 1639 and 3460 cm^–1^ are associated with O–H or H–O–H.^46^ The materials after metal deposition exhibited a much more intense band associated with hydroxyl-group vibrations than Alu C-805 did; this might be attributed to the removal of hydrophobic groups, resulting in a more hydrophilic material. The presence of C–OH species formed during carbon-layer deposition can account for the peak’s intensification. The Alu C-805 also showed a broad, low-intensity peak at about 1100 cm^–1^, commonly attributed to the asymmetric Si–O–C or Si–O–Al vibration. After Ni nanoparticle formation at high temperatures, the band was also observed. Two additional bands at 2860 and 2980 cm^–1^, attributed to C–H stretching from the hydrophobic alkyl group, are observed for Alu C-805.^47,48^

After thermal treatment in Ar at 400 °C, these bands disappear, indicating substantial transformation of the surface organic functionalities during Ni-based nanoparticle formation. This behavior may be attributed not only to the decomposition of organosilane or ligand-related groups, but also to their pyrolytic transformation, which involves cross-linking and carbonization processes that form a thin carbonaceous layer on the support surface. This layer is largely depleted of aliphatic C–H moieties, which explains the loss of the corresponding IR bands and may contribute to the encapsulation or stabilization of Ni nanoparticles. The Ni_P spectrum exhibits three intensity peaks at approximately 1129, 720, and 481 cm^–1^, corresponding to P–O stretching vibrations.^49^

TG analysis was used to quantify mass loss and assess the organic matter loading deposited on the alumina surface. Figure S2 and Table S1 show the results. Mass loss varies from 5% for Alu C-805 and Ni_Nit to 18% for Ni_P. The distribution of chemical elements on the solid surface was also examined using X-ray photoemission spectroscopy (XPS), and the results are displayed in Figure S3. The results show the same trend as in TGA; however, in XPS, the analysis depth is limited to the surface (<5 nm).

Figure 2 shows high-angle annular dark-field scanning transmission electron microscopy (HAADF-STEM) images of Ni-based catalysts. The corresponding particle-size distribution histograms are shown in Figure S4, and the average particle sizes are summarized in Table 1. The particle size distribution varies considerably, with an average particle size of 10 nm (in the absence of a ligand) and a much smaller size of 4.8 nm (with citric acid as the ligand). This might be related to the higher mobility of Ni ions on the support surface than that of Ni complexes, leading to easier particle growth during pyrolysis. In the absence of complexation, Ni ions are likely more mobile, promoting aggregation and particle growth during thermal treatment. In contrast, Ni-ligand complex formation stabilizes the metal species and limits their mobility at elevated temperatures, resulting in smaller, more uniformly dispersed nanoparticles.

**Figure 2:**
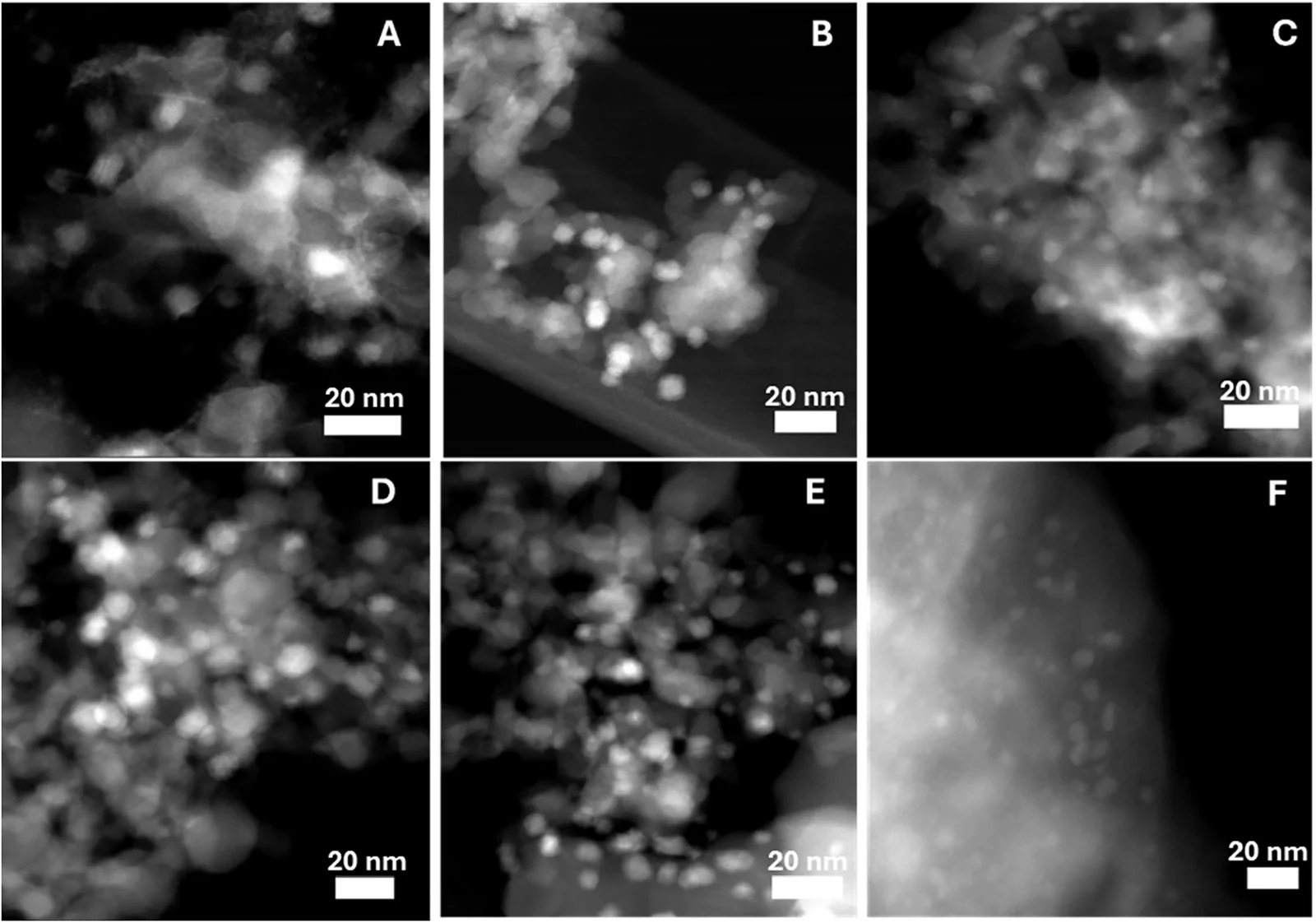
HAADF-STEM images (A) Ni_Nit; (B) Ni_URO; (C) Ni_CA; (D)­Ni_Etdi; (E) Ni_Mel; (F) Ni_P.

Figure 3A shows the chemical maps (Al, O, and Ni) of Ni_CA, derived from energy-dispersive X-ray spectroscopy (EDX) combined with a STEM image. The image shows Ni nanoparticles within the scanned area, visible as bright, round particles. Moreover, elemental mapping visualizes the quasi-porous architecture of the alumina–carbon support, with dark, circular features evident. These features do not correspond to the intrinsic porosity of the material but rather arise from interparticle voids formed between aggregated support grains. The chemical maps of other samples (Ni_Nit, Ni_URO, and Ni_Mel) are shown in Figure S5. The chemical mapping also indicates the formation of larger Ni particles for Ni-Nit, corroborating the XRD analysis.

**Figure 3:**
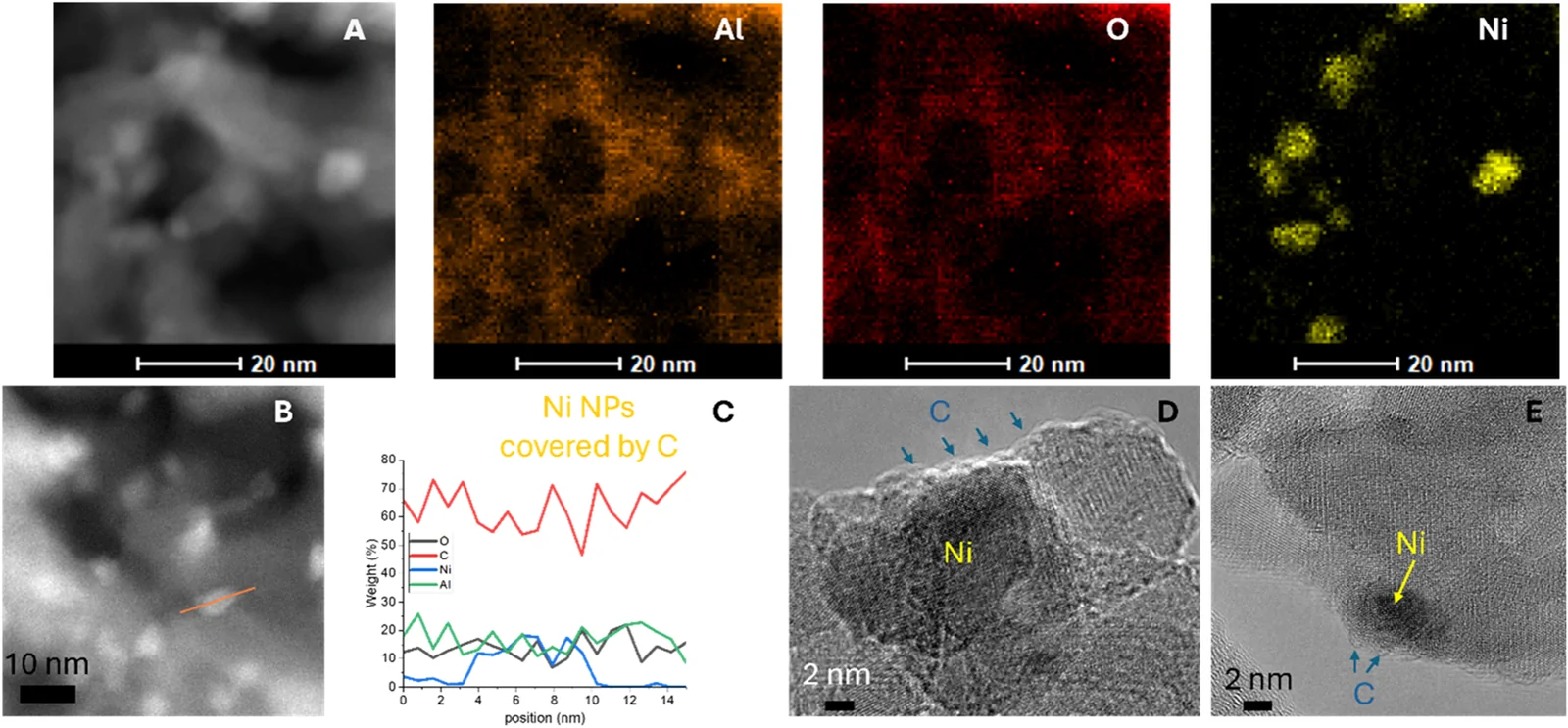
Microscopic results obtained for the Ni_CA sample: (A) HAADF- STEM image combined with chemical mapping, (B, C) HAADF-STEM image and its corresponding EDX line scan; (D, E) High-resolution TEM.

Figure 3B shows a STEM image along with an EDX line-scan profile of Ni_CA. The overlap of the Ni and C signals along the scan line indicates their spatial localization, suggesting an intimate association between nickel particles and carbon species. Consistent results were obtained from a different region of the sample (Figure S6), confirming a uniform distribution of carbon near the metal particles. High-resolution TEM images also indicate the formation of very thin, amorphous carbon layers covering the surfaces of Ni particles. Figure 3D,E show a wave-shaped carbon structure (indicated by a blue signal) on the edges of metal particles (indicated by yellow). The thickness of this carbon overlayer is estimated to be 0.5–1 nm.

Raman spectroscopy was used to assess the degree of graphitization and disorder in the C-layer present in the samples. Figure S7 shows the spectrum; two major peaks are observed at 1373 and 1555 cm^–1^, corresponding to the D and G Bands, respectively. The D band is associated with defective components in the carbon structure, including topological defects, additional functional groups, and vacancies. The G-band is related to graphitized structures. The *I*_D_/*I*_G_ ratio is commonly used to characterize the degree of graphitization; a ratio above 1 indicates a highly defective amorphous carbon structure.^50,51^ Ni_CA exhibits an *I*_D_/*I*_G_ of 1.01, indicating the formation of a highly defective structure, typically at low pyrolysis temperatures (below 1000 °C).

Temperature-programmed desorption coupled with mass spectrometry (TPD-MS) was used to characterize gas release from the samples in helium up to 800 °C, as shown in Figure S8. The evolution of CO during temperature-programmed experiments can be used to identify the functional groups present on carbonaceous materials. CO release at relatively low temperatures (<300 °C) is usually associated with the decomposition of aldehyde or ketone groups; 320–600 °C decomposition of anhydrides; phenols at 500–750 °C; and carbonyl or quinone groups (650–950 °C). In the case of CO_2_ release, carboxylic acids normally decompose at 100–400 °C, lactone groups at 200–600 °C, and at 400–900 °C.^52–55^

For *m*/*z* = 28, Ni_CA exhibits a broad peak from 320 °C to 800 °C, centered at 482 °C, suggesting a carbon layer with diverse oxygenated functional groups, especially aldehydes, ketones, and anhydrides. In Ni_URO, a broad peak was also observed, but it was centered at a higher temperature (610 °C), indicating a higher concentration of phenolic, carbonyl, and quinone groups. Monitoring *m*/*z* = 44, which corresponds to CO_2_ and N_2_O, Ni_URO and Ni_CA show a very broad peak from 150 to 650 °C (Ni_URO) and 150–550 °C (Ni_CA). Ni_CA shows a sharper peak, with the highest point around 450 °C, attributed to anhydrides. The combined TPD_MS analyses, together with the other analyses (Raman, XPS, and XRD), indicate a poorly graphitized carbon structure and a highly functionalized carbon layer with a diversity of oxygenated functional groups (in some cases, N-groups as well), as expected for the low carbonization temperature (below 800 °C) where aromatization reactions are limited.

Figure 4A shows high-resolution C 1s XPS. All materials exhibited a broad peak between 282 and 291 eV. The spectra could be deconvoluted into 3–5 regions, with peaks at 284.3, 285.2, 286.7, 287.9, and 289.0 eV corresponding to Csp^2^, Csp^3^, C–O or C–N, C=O, and COO.^56,57^ This is also consistent with the FTIR and TPD-MS data, which show an increase in the O–H band intensity in the solids after C-layer deposition and the presence of multiple oxygenated functional groups. The C–O group could also increase the support’s hydrophilicity through H-bonding with H_2_O.

**Figure 4:**
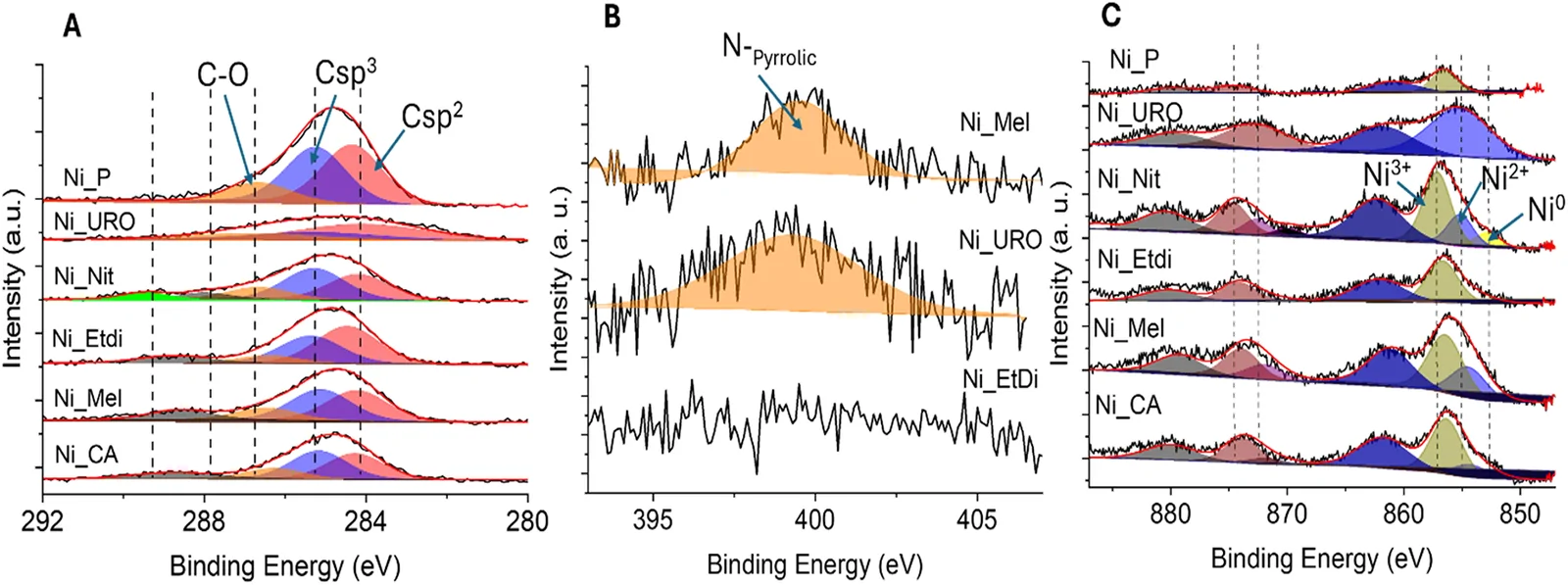
High-resolution XPS spectra of (A)C 1s; (B) N 1s, and (C) Ni 2p.

High-resolution XPS N 1s spectra of C layers deposited on Ni/alumina are shown in Figure 4B. The peak was centered at approximately 400.1 eV and deconvoluted into a single N chemical species, N-pyrrolic. N-pyrrolic is classified as a soft base; thus, this species exhibits an electron-donating profile.^58^ The nitrogen content on Ni_Etdi was below the detection limit, likely because ethylene diamine has a low boiling point, making its fixation on the alumina surface difficult. Figure S9 shows the P 2p high-resolution spectra, which exhibits a relatively broad peak between 131 and 137 eV. The spectrum was deconvoluted into two components centered at 133.78 and 134.7 eV, which are commonly associated with phosphorus atoms bonded to oxygen.^59,60^

High-resolution Ni 2p XPS was also measured and is shown in Figure 4C. Two peaks at 855 and 872 eV were observed in all samples and are associated with Ni 2p_3/2_ and Ni 2p_1/2_, respectively. The extra peaks at 862.4 and 879.9 eV are the corresponding satellite peaks.^61,62^ The peak at 855 eV could be deconvoluted into 2 or 3 chemical environments, depending on its width. The centers of these peaks are approximately 852.7, 855.12, and 857.2 eV, associated with Ni^0^, Ni^2+^, and Ni^3+^, respectively. The high-resolution Ni XPS spectra vary substantially with the ligands used during the synthetic procedure. Ni_Nit shows three distinguishable peaks. However, materials synthesized from Ni-complex precursors show a shift of the Ni^3+^ peak to lower binding energy, suggesting a mixed Ni^δ+^ state between Ni^2+^ and Ni^3+^.^61^ N-species present on C-layers can act as Lewis bases, donating an electron pair to the metal center, thereby forming an interface between the metal and the support (Ni_Mel and Ni_URO). In Ni_CA, carbonyl or hydroxyl groups could act as electron donors. These results demonstrate the influence of the carbon layer on the nickel electronic structure and the strong interaction between them.

Thermally programmed reduction under hydrogen (TPR-H_2_) analyses are shown in Figure S10. Ni_P shows very little H_2_ uptake, suggesting that most Ni ions are not accessible to H_2_ molecules, which is also in line with XPS Ni 2p, which shows a much lower peak intensity than in the other solids. Another possibility is that Ni is hardly reducible due to specific interactions within the material. The Ni_Nit and Ni_Etdi samples exhibit a broader reduction temperature range, from approximately 320 to 580 °C, commonly referred to as the reduction of the nickel oxides supported on alumina or carbon with a moderate support interaction.^63^ Moreover, a broader particle-size distribution, as observed by STEM, also drives the formation of broad peaks in TPR-H_2_.^64^ This also corroborates the observation that ethylenediamine volatilizes during pyrolysis, likely due to its low boiling point, resulting in a broad Ni particle-size distribution, comparable to Ni_Nit. On the other hand, Ni_Mel and Ni_URO exhibit a much sharper profile centered at 463 °C, commonly associated with nickel oxide deposited on N-doped carbon.^37,65^ These profiles also indicate a much narrower Ni particle-size distribution consistent with STEM images.

Ni_CA exhibits a three-stage profile: a weak initial peak centered at 400 °C, followed by a negative peak, and a third, more intense peak. This negative peak at 465 °C may be related to the reduction of oxygenated species on the carbon surface; however, the nature of the interaction between H_2_ and the carbon remains unclear. Some authors suggested a reaction between carbon–oxygenate species, followed by the thermal desorption of oxygen surface complexes.^66,67^ This also correlates with TPD-MS analysis, which indicates the desorption of some functional groups under the same temperature range. A TPR-H_2_ coupled to a mass spectrometer was also used to monitor CO release (*m*/*z* = 28; Figure S11). CO release indicates partial reduction of the carbon layer. Another possible phenomenon is the desorption of H_2_ from the nickel surface (strongly chemisorbed), which can cause atomic restructuring of Ni@C particles.^68^ The TPR-H_2_-MS profile (Figure S11) also shows hydrogen release from the sample, indicating this restructuring phenomenon, in which a less functionalized carbon structure is present on the nickel surface. The last peak, centered at 530 °C, is attributed to the reduction of nickel oxides, which interact strongly with the support.^69^

The Ni-based materials were tested as catalysts for the hydrogenation of CO_2_ under atmospheric pressure in a fixed-bed reactor at a CO_2_/H_2_ (v/v) ratio of 1:4. The comparison of the catalytic activity of the solids at different temperatures is shown in Figure 5(a) and Table 2. The solid catalysts coated with a carbon layer (both N-doped and undoped) exhibit consistent temperature-dependent behavior. The highest catalytic activity is observed at ≥ 440 °C, whereas the maximum methane selectivity occurs at lower temperatures (∼370 °C). In all cases, only two products were observed: CH_4_ (the major product) and CO (the side product). This trend is in good agreement with the results reported by Wu et al. for Ni nanoparticles confined in microporous graphene-like carbon, where CO_2_ conversion increases with temperature up to 400–450 °C, while CH_4_ selectivity reaches a maximum around 400 °C and then gradually decreases.^70^ This behavior can be rationalized by considering the interplay between kinetics and thermodynamics.

**Figure 5:**
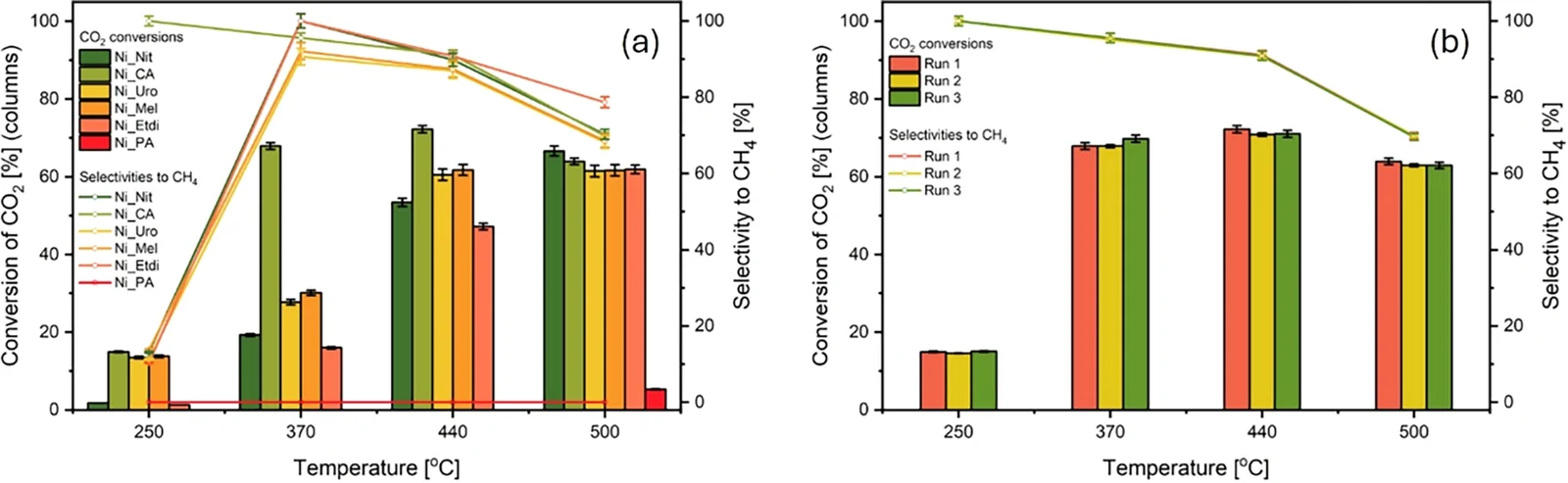
(a) CO_*2*_ conversion under different temperatures (bars) and the selectivity to methane (line-spheres) for the synthesized materials; (b) Recyclability of Ni_CA catalyst under different temperatures.

**Table 2:** Catalyst Properties, Catalytic Process Parameters and Calculated Reaction Parameters for Methane Formation, Normalized to Total Nickel Content in the Catalysts (r_m_) and to Catalysts’ Surface Areas (r_S_), Together with Turnover Frequencies (TOF), Calculated from Catalytic Results at 370 °C^h^

		Catalyst properties		Catalytic process parameters					Catalytic data			Calculated catalytic parameters			
Catalyst		Ni loading [wt %]	Ni particle size [nm]	Catalyst mass [g]	Concen-tration of CO_2_ [-]	Gas mixture composi-tion	Gas flow [L·min^–1^]	Process tempera-ture [°C]	CO_2_ conver-sion [%]	CH_4_ selecti-vity [%]	CH_4_ yield [%]	r_m_ (mmol_CH4_·g_Ni_^–1^·min^–1^)^a^	r_S_ (μmol_CH4_·m^–2^·min^–1^)^b^ ^a^	TOF_CH4_·10^–4^ (s^–1^)^c^	refs
Ni_Nit		5.1	10.1 ± 2.2	0.15	0.04	H_2_:CO_2_:He4:1:7.5	0.05	370	49	95	47	505.3	2.65	1.43	This work
Ni_Etdi		4.1	8.7 ± 2.2						16	100	16	213.8	1.01	0.61	
Ni_URO		4.4	6.3 ± 1.4						27	91	24	306.3	1.43	0.87	
Ni_Mel		4.6	6.4 ± 1.3						29	92	26	311.1	1.75	0.88	
Ni_P		3.9	7.7 ± 2.3						0	0	0	0	0	0	
Ni_CA	Run 1	4.1	4.8 ± 1.3						70	96	67	921.9	4.38	2.62	
	Run 2								70	95	67	923.7	4.39	2.62	
	Run 3								68	96	65	900.2	4.28	2.56	
Ni(5%)@NC/SiO_2_ ^d^		5	17 ± 1.4	0.015	0.02	H_2_:CO_2_:Ar4:1:45	0.1	350	15	8	1	133.0	n.d.	0.38	^37^
Ni(5%)@C/SiO_2_ ^d^			n.d.						13	17	2	245.0	n.d.	0.70	
Ni(5%)^imp^/SiO_2_ ^d^			5–60						16	25	4	443.5	n.d.	1.26	
Ni(5%)/MGC^e^		5	n.d.	0.1	0.166	H_2_:CO_2_:N_2_ 4:1:1	0.06	370	32	60	19	1590.0	0.37	4.52	^70^
Ni(15%)/MGC^e^		15							49	95	47	1285.0	1.16	3.65	
Ni(10%)/Al_2_O_3_ commercial nonporous support^f^		10	5.2	0.1	0.15	H_2_:CO_2_:N_2_ 12:3:5	0.017	370	40	96	38	399.9	2.72	1.14	^71^
NKW-2 V commercial Ni catalyst^g^		13.3	n.d.	0.6	0.2	H_2_:CO_2_4:1	0.1	350	63	100	63	656.4	-	1.86	^72^

a Specific reaction rate of CH_4_ formation related to the total Ni content in the catalyst (r_m_) was calculated assuming ideal gas behavior at standard conditions. For the catalysts studied in this work the calculations were done at selected temperature 370 °C basing on the gas flow rate (v = 0.05 L.min^−1^), 4 vol % initial concentration of CO_2_ in the gas flow (volume fraction C_CO2_ = 0.04 (−)), and CH_4_ yield at selected temperature (Y_CH4_ (−)), and the mass of Ni in the catalyst (m_Ni_ (g)) calculated using elemental analyses data (Table 1) using the following equation: r_m_(mmol_CH4_·g_Ni_^–1^·min^–1^) = ((Pv/*RT*)·Y_CH4_ (−)·C_CO2_ (−))/m_Ni_ (g) = ((1013.25­(hPa)·0.05­(L·min^–1^))/(83.14­(hPa·L·mol^–1^·K^–1^)·298.15 (K))·0.04·Y_CH4_ (−)·10^3^)/(m_Ni_ (g)) = (8.31·10^–2^·Y_CH4_)/m_Ni_. Parameters related to the literature data were calculated the same way, using appropriate process parameters provided in the cited papers.

b Specific reaction rate of CH_4_ formation related to the surface area of the catalyst (r_S_) was calculated at selected temperature using a similar equation as for r_m_, with taking into account the catalyst mass (m_cat_(g)), and specific surface area calculated using BET method (*S*_BET_ (m^2^·g^–1^)): r_S_(μmol_CH4_·m^–2^·min^–1^) = (8.31·10^–2^ (mmol_CH4_·min^–1^)·Y_CH4_ (−)·10^3^)/(m_cat_ (g)·*S*_BET_(m^2^·g^–1^)) = 83.1/(m_cat_ ·*S*_BET_). Parameters related to the literature data were calculated the same way, using appropriate process parameters provided in the cited papers.

c Turnover frequency of CH_4_ formation (TOF) at selected temperature was calculated based on r_m_(mol_CH4_·min^–1^.g_Ni_^–1^) by normalization of the reaction rate of CH_4_ formation (in mol_CH4_·g_Ni_^–1^.s_^-1^_) to the total number of moles of Ni in the catalyst: r_m_(mol_CH4_·g_Ni_^–1^·min^–1^)/(M_Ni_(g·mol^–1^))=TOF (min^–1^)/60.

d Ni­(5%)@NC/SiO_2_ and Ni(5%)@C/SiO_2_ catalysts were composed of Ni nanoparticles covered with N-doped carbon, or nondoped carbon shells, respectively, and deposited on commercial HDK T40 silica support. Ni(5%)^imp^/SiO_2_ catalyst was obtained by incipient wetness impregnation of nickel acetate on the same support. The data taken for calculations were obtained at 350 °C.^37^

e 5 and 15 wt. Ni were deposited on MGC - microporous graphene-like carbon support. Specific surface area of the catalysts were *S*_BET_ = 2413.7 and 1655 m^2^.g^–1^, respectively.^70^

f Specific surface area of studied 10 wt % Ni deposited on commercial nonordered alumina was: *S*_BET_ = 146.9 m^2^.g^–1^ ^71^

g Catalyst studied in that work was commercial granulated nickel methanation catalyst NKM-2 V (produced by Novomoskovsk Institute of Nitrogen Industry, Russia). Composition of the catalyst obtained by AAS: 23.4 wt % of Ni, 13.3 wt % of Al, 7.36 wt % of Ca, 0.57 wt % of Si.^72^

h Data are compared with parameters estimated from literature data.

Ni_P was inactive as a catalyst for CO_2_ hydrogenation; this solid has a very low surface area and no pores, as shown by N_2_ physisorption isotherms. TPR-H_2_ of Ni_P shows very little H_2_ uptake, suggesting that most Ni ions are not accessible to H_2_ molecules, which is also in line with XPS Ni 2p, which shows a much lower peak intensity than in the other solids. The TEM image also suggested that the Ni particles are inserted into a much larger support structure, which is not characteristic of AluC-805, suggesting low accessibility of reactants to the metal center.

At lower temperatures, the hydrogenation pathway toward methane is kinetically favored on highly dispersed Ni sites, particularly when stabilized within a confined carbon matrix, leading to high CH_4_ selectivity. However, as the temperature increases, the intrinsic reaction rate rises (enhancing overall CO_2_ conversion), but the thermodynamic limitation of the exothermic Sabatier reaction becomes increasingly significant. Consequently, above 450–500 °C, methane formation is disfavored, and the reverse water–gas shift (RWGS) reaction, which is endothermic, becomes more competitive. As a result, at elevated temperatures (>500 °C), the product distribution shifts toward CO formation, reducing methane selectivity despite relatively high CO_2_ conversion. While Wu et al. linked catalytic performance to confinement of Ni nanoparticles within a microporous carbon framework, this effect can be excluded here. The carbon layer has porosity arising mainly from interparticle voids rather than a defined porous network.

The product distribution obtained at atmospheric pressure is influenced not only by intrinsic catalytic properties but also by thermodynamic limitations, particularly at elevated temperatures. The formation of CH_4_ via the exothermic CO_2_ methanation reaction becomes progressively less thermodynamically favorable with increasing temperature, whereas the endothermic RWGS reaction is favored at higher temperatures. Consequently, the increasing contribution of CO at elevated temperatures cannot be attributed exclusively to changes in the intrinsic catalytic properties.

Interestingly, at 500 °C, the CO_2_ conversions and CH_4_ selectivity obtained over almost all investigated catalysts become very similar, despite the differences observed at lower temperatures. This convergence suggests that, at high temperature, the attainable product distribution is increasingly constrained by thermodynamics under the applied atmospheric-pressure conditions. Nevertheless, the distinct temperature-dependent changes in conversion and CH_4_ selectivity observed for the individual catalysts indicate that their intrinsic catalytic properties still play an important role, particularly at lower and intermediate temperatures. Thus, differences in catalytic performance in this temperature range should be considered in terms of both catalyst-dependent reaction kinetics and progressively increasing thermodynamic limitations at higher temperatures.

Arpini et al. demonstrated that encapsulation of Ni nanoparticles within N-doped carbon shifts CO_2_ hydrogenation selectivity from CH_4_ toward CO at 350–450 °C relative to undoped and bare Ni catalysts.^37^ Based on in situ DRIFTS, this behavior was attributed to an increased fraction of linearly adsorbed CO species associated with the RWGS pathway, whereas bare Ni favored multibound CO species prone to dissociative hydrogenation and methanation. In the present study, this effect is less pronounced: N-doped carbon-supported catalysts exhibit only a slight decrease in CH_4_ selectivity at 370 and 440 °C, despite significantly lower methane formation rates, and CH_4_ remains the dominant product under all conditions. Consistently, the behavior reported for metallic Ni by Arpini et al. may also account for the higher specific reaction rate and turnover frequency for methane formation observed for the bare Ni_Nit catalyst compared to most of the encapsulated systems investigated here.

These differences likely arise from structural factors, including smaller Ni particle size and lower carbonization temperature, which limit the formation of carbide-like phases and weaken metal–support electronic interactions. Such interfacial features modulate the electronic structure of Ni and, consequently, CO adsorption geometry. Electronically perturbed Ni surfaces preferentially stabilize linear CO, favoring RWGS, whereas metallic Ni ensembles promote bridging CO species that facilitate C–O bond cleavage and methanation. Thus, variations in metal–support interactions govern the RWGS-methanation balance, and the limited electronic perturbation in the present catalysts sustains methane as the primary product.

The results are consistent with those reported by Visser et al., who did not observe a pronounced effect of carbon functionalization on the CO_2_ hydrogenation selectivity of Ni catalysts supported on modified carbon under 30 bar.^73^ In that study, the Ni particle sizes were nearly identical across all samples prior to reaction, effectively eliminating particle size effects on catalytic performance. Instead, the role of support was primarily reflected in catalyst stability: surface functionalization significantly influenced nanoparticle sintering, with oxygen-functionalized carbon providing the highest resistance to particle growth among the investigated systems. Thus, in terms of product distribution, the contribution of the carbon support appears to be limited when structural parameters are well controlled.

A different trend is observed in CO_2_ conversion, where more pronounced differences among the catalysts emerge. From 250 to 440 °C (Figure 5), systems containing encapsulated Ni nanoparticles exhibit significantly higher activity than the noncovered Ni_Nit and Ni_Etdi references, for which XPS and TGA indicate only a marginal increase in carbon content. Among all materials, Ni_CA displays the highest activity at 370 and 440 °C. This enhanced performance can be attributed to a combination of factors, including (i) a smaller average Ni particle size and (ii) a synergistic interaction between Ni nanoparticles and the carbon overlayer derived from citric acid, which likely modifies the local electronic and geometric environment of the active sites.

The observed size–activity relationship is consistent with previous reports.^22,74,75^ Simons et al. reported that while the conversion of CO_2_ to CO is insensitive to Ni particle size, methanation rates reach a maximum for particles around 5 nm, with a sharp decline in CH_4_ selectivity observed for particles below 4 nm.^75^ This aligns with the present results, where Ni_CA, characterized by an average particle size of 4.8 nm, exhibits the highest catalytic activity. This behavior can be rationalized based on the mechanistic insights of Simons et al.^74^ who demonstrated that particle size mainly influences the CO hydrogenation step, whereas CO_2_ activation and initial CO formation proceed similarly across a wide range of Ni sizes. The reduced methanation activity on sub-4 nm particles was attributed to insufficient stabilization of CO* intermediates at low-coordination sites, thereby impeding further hydrogenation to CH_4_. In contrast, particles in the 4–6 nm range provide a more favorable balance between terrace and step sites, enabling efficient conversion of CO* to methane. Furthermore, the negligible variation in CO selectivity across the studied catalysts can be rationalized by the Ni particle size distribution remaining predominantly above 4 nm, a regime in which particle-size effects on CO formation are minimal.

Another factor that may influence catalytic performance and stability is the presence of a carbon-derived layer on the catalyst surface, which can modify CO_2_ adsorption through both dipole–dipole interactions involving residual oxygen-containing species and π–π interactions with graphitic domains, as suggested by *ab initio* studies on aromatic model systems.^76^ Such interactions are particularly relevant at relatively low temperatures (≤400 °C), where weakly bound adsorption complexes may contribute to CO_2_ activation.

In carbon-supported Ni catalysts, acid treatments and oxygen functionalization have been shown to strongly affect metal–support interactions and, consequently, catalytic behavior in reforming and hydrogenation reactions.^77,78^ However, H_2_-TPR results indicate that for the Ni_CA catalyst, the carbon layer undergoes partial reductive deoxygenation during the prereduction step, thereby decreasing the concentration of oxygen-containing functionalities. Importantly, this process does not merely eliminate surface functionalities but can also modify the electronic structure of the carbon surface, increasing its electron density through the removal of strong electron-withdrawing oxygen groups and the partial formation or exposure of less oxidized species, such as alcohol-, or carbonyl-type groups, as well as defect-rich graphitic domains. As a result, the surface may exhibit an increased apparent basicity, which is more favorable for CO_2_ adsorption and activation.^79^ Consequently, the reduction-induced restructuring of the carbon layer likely modulates the balance between CO_2_ adsorption and subsequent hydrogenation steps in the methanation reaction.

Comparing the specific reaction rates for methane formation (Table 2) with values reported in the literature, it can be concluded that the prepared materials exhibit comparable catalytic performance, with the Ni_CA sample showing the highest activity in both specific rate and intrinsic catalytic efficiency. It is noteworthy that in the reference study,^73^ the reaction was carried out under significantly higher pressure (approximately 30 times higher than in the present work), which inevitably led to a substantially increased surface concentration of reactants and, consequently, more favorable kinetic conditions. Taking this into account, the observed performance of the Ni_CA catalyst under considerably milder reaction conditions further highlights its intrinsic activity and the beneficial role of its optimized physicochemical properties, which enable efficient CO_2_ activation and subsequent hydrogenation even at lower reactant chemical potentials.

The recyclability of Ni_CA was also evaluated at different reaction temperatures (Figure 5b). The catalyst maintains both activity and selectivity over three consecutive cycles conducted up to 550 °C, indicating very good operational stability under the applied methanation conditions. Postreaction characterization (Figures S12 and S13) confirms that the catalyst’s structural integrity is largely preserved. The XRD patterns of the spent sample show no emergence of distinct Ni reflections, suggesting that Ni nanoparticles remain highly dispersed and below the detection limit for bulk crystallite growth. This is consistent with HAADF-STEM analysis, which reveals only a slight increase in the average particle size from 4.8 to 5.0 nm, indicating negligible particle growth during reaction.

The stability of Ni_CA was also tested under long-term on stream conditions (50 h), typical for industrial catalyst tests. The result is shown in Figure S14; the catalyst is extremely stable, and no decrease in catalytic activity was observed; moreover, selectivity did not change. We examined the catalyst structure by TEM; Figure S15 shows that most particles remain in the 5 nm range, as in the fresh catalyst.

Such stability can be rationalized by the presence of the carbon-derived layer, which, as discussed above, may act as a stabilizing matrix that limits the surface mobility of Ni species and suppresses agglomeration. In addition, partial deoxygenation of the carbon surface during prereduction likely yields a more defect-rich, electronically modified support, which can enhance metal–support anchoring strength and further inhibit particle migration. Similar stabilization effects of carbonaceous or oxygen-functionalized supports on Ni dispersion have been reported in the literature, in which strong metal–support interactions and confinement within carbon matrices significantly reduce tendencies for metal particle growth in hydrogen-rich environments.^80,81^

## Experimental Section

Chemical and characterization data are displayed in the Supporting Information.

### Synthesis of Solid Catalysts

The methodology was adapted from protocols reported by the Beller and Rossi groups.^37,82–84^ The solid catalysts were prepared by immobilizing nickel complexes on alumina (AluC-805), evaporating the solvent under vacuum, and subsequently carbonizing the resulting solids.

In a typical procedure, 0.69 mmol of Ni­(NO_3_)_2_·6H_2_O and 2.1 mmol of ligand were dissolved in 20 mL of ethanol under vigorous stirring, then allowed to react for 16 h at 40 °C. The solution was then transferred to a round-bottom flask containing 810 mg of alumina. The suspension was dried under vacuum at 60 °C. The dried solid was pyrolyzed at 400 °C for 2 h under argon at a heating rate of 3 °C. min^–1^.

### Catalytic Test

A 150 mg portion of powdered catalyst was loaded into a U-shaped quartz reactor and secured with a quartz wool plug. The reactor was installed in a fixed-bed flow system placed inside a temperature-controlled tubular furnace. Before catalytic measurements, the sample was reduced in situ at 550 °C for 1 h at a heating rate of 3 °C min^–1^ under a flow of pure hydrogen (20 mL·min^–1^).

After reduction, the reactor was cooled to room temperature under a hydrogen atmosphere, and the feed was subsequently switched to a reaction mixture containing CO_2_ (2 mL·min^–1^), H_2_ (8 mL·min^–1^), and He as the balance gas (40 mL·min^–1^). Once stable gas flow and baseline conditions were established, the catalytic test was conducted under continuous flow while heating from 30 to 550 °C at a linear rate of 3 °C·min^–1^.

The reaction products were analyzed online using a PerkinElmer Clarus 500 gas chromatograph equipped with a Carboxen-packed column and both thermal conductivity (TCD) and flame ionization (FID) detectors. The concentrations of reactants and products were recorded at 10 min intervals under dynamic temperature-programmed conditions. For clarity and direct comparison, catalytic performance is discussed at selected characteristic temperatures.

The catalytic durability test was performed using the same experimental setup as the temperature-dependent catalytic tests. Before the measurement, 150 mg of the 5% Ni_CA catalyst was placed in the reactor and reduced in situ at 550 °C for 1 h under a pure H_2_ flow (20 mL·min^–1^), using a heating rate of 3 °C·min^–1^. The system was then cooled down to room temperature, and the catalyst was flushed with a CO_2_/H_2_/He mixture at flow rates of 2, 8, and 40 mL·min^–1^, respectively. Subsequently, the sample was heated to 370 °C at 3 °C·min^–1^ under the gas mixture flow and maintained at this temperature. The catalytic performance was monitored for 50 h, with measurements recorded at 1 h intervals.

## Conclusions

A group of nickel-based catalysts was synthesized by thermal decomposition of Ni complexes under argon over an Alu-C-805 alumina support. The combination of high-resolution TEM with EDS measurements suggests the formation of a thin carbon layer around the nickel particles. Thanks to the selection of the ligand, the particle size could be tuned, and the C layer structure could be modified by introducing dopants such as N. When phytic acid was used as a ligand, the formation of alumina phosphate was observed, and the obtained catalyst was inactive. The material containing Ni nanoparticles encapsulated by a carbon overlayer exhibits higher performance than the “bare” Ni particles at low temperatures. The significantly higher activity of the Ni_CA catalyst can be explained by the smaller Ni particle size formed during decomposition of the Ni–ligand complexes, as well as by a synergistic interaction between the ultrathin carbon overlayer and the Ni nanoparticles, which may modify the surface electronic properties and enhance the accessibility and reactivity of the active sites. XPS spectra reveal a modification in the electronic properties of the carbon-encapsulated Ni nanoparticles, characterized by a mixed Ni^2+^/Ni^3+^ oxidation state. This observation demonstrates a strong interaction between the Ni particles and the surrounding carbon layer, leading to partial electron transfer and surface reorganization. Moreover, the catalyst exhibits excellent thermal stability, attributable to the stabilizing effect of the carbon overlayer, which inhibits particle growth under reaction conditions.

## Supplementary Material



## Notes

Description of (1) chemicals and materials, (2) equipment used to characterize the materials; (3) Complementary results: TEM image (Figure S1); TGA analyses (Figure S2); organic matter content (Table S1); surface composition (Figure S3); particle size distribution (Figure S4); EDS-STEM images (Figure S5); HAADF-STEM image with EDX line scan (Figure S6), Raman spectrum (Figure S7); TPD-MS analyses (Figure S8); high-resolution P 2p XPS analysis (Figure S9); TPR-H_2_ analyses (Figure S10), TPR-H_2_-MS analyses (Figure S11); XRD pattern of spent catalyst (Figure S12); HAADF-STEM and the particle size distribution of spent catalyst (Figure S13); Stability test (Figure S14); TEM analysis of spent catalyst (Figure S15) (PDF)
