# Supplementary material for: Boosting CO2 Hydrogenation Activity by Carbon Coating over a Nickel Catalyst
Source: ACS Omega. 2026 Oct 14;11(42):65442–53. doi: 10.1021/acsomega.6c08194 (PMC13717641; doi:10.1021/acsomega.6c08194)
Supplement: Supplementary file 1 [file ao6c08194_si_001.pdf]

Supporting Information

Boosting CO_2 hydrogenation activity by carbon coating over a nickel catalyst

Rafael L. Oliveira* [a], Karolina A. Ledwa [a], Olga Chernyayeva [b], Krzysztof Matus [c]

[a] Institute of Low Temperature and Structure Research of the Polish Academy of Sciences, Wroclaw, 50-422, Poland

[b] Institute of Physical Chemistry of the Polish Academy of Sciences, Warsaw, 01-224, Poland

[c] Materials Research Laboratory, Silesian University of Technology, Gliwice, 44-100, Poland

Corresponding author

e-mail: r.oliveira@intibs.pl

Chemicals and Materials

Alu_C 805 was purchased from Evonik Industries. Citric acid (99.5%) was purchased from PolAura. Urotropina (99%) was obtained from Firm ChemPur. Melamine (99%) and Ethylene diamine (99.5%) were purchased from Sigma-Aldrich. H_2 99.999% was purchased from Messer Helium, 99.999 % was obtained from PGNiG S. A Carbon dioxide (99.999%) and compressed air were purchased from Linde.

Characterization

The crystalline structure of the synthesized materials was determined using an X'Pert PRO PANalytical powder diffractometer with Cu Kα radiation. The X'Pert HighScore Plus program was used to display and analyze diffraction patterns. Data were collected over 2θ = 20–80°.

N_2 physisorption analysis was performed at -196.15 ºC using a Micrometrics ASAP 2020 automated system. The samples were dried under vacuum at 150 °C for 6 h prior to measurement. The average pore size of the materials was calculated from the adsorption branch of the isotherm using the Barrett–Joyner–Halenda (BJH) method.

Thermogravimetric analysis (TGA) was performed on a Derivatograph TG-DTA (MOM, Budapest). The samples (~100 mg) were heated from room temperature to 800 °C at 5 °C/min under air, then held at 800 °C for 1 h.

Microstructural investigations were performed at an accelerating voltage of 300 kV using a STEM TITAN 80–300 (FEI). The instrument featured a Schottky-type FEG source and was equipped with a CEOS CETCOR probe and Cs corrector, enabling acquisition of aberration-corrected, high-resolution images. Particle size was measured using ImageJ, and the size distribution was analyzed by generating a histogram. The microstructure of Alu C 805 and spent catalyst (50h durability test) was investigated using a Philips CM-20 SuperTwin TEM operating at 160 kV.

The XPS spectra were recorded with a PHI 5000 VersaProbeII™ scanning ESCA Microprobe (ULVAC-PHI, Japan) using monochromatic Al-Kα radiation (hν = 1486.6 eV) from an X-ray source operating at 100 µm spot size, 25 W power, and 15 kV acceleration voltage, area of 500μm^2. The high-resolution (HR) XPS spectra were acquired using a hemispherical analyzer at a pass energy of 23.5 eV, an energy step size of 0.1 eV, and a photoelectron take-off angle of 45° relative to the surface.

The obtained XPS spectra were analyzed in CasaXPS software (v.2.3.26PR1.0, Casa Software Ltd, United Kingdom) using the native hardware sensitivity factors. The binding energy scale was referenced to the C1s peak at 284.8 eV.

The Fourier transform infrared (FTIR) spectra in the region 4000–400 cm^{-1} were recorded using KBr pellets with a Thermo Scientific Nicolet Apex FTIR spectrometer. Raman spectra were recorded in the 100–3500 cm–1 range using a Renishaw InVia Raman spectrometer equipped with a confocal DM 2500 Leica optical microscope, a thermoelectrically cooled CCD as the detector, and an Ar laser operating at 514.5 nm. The laser beam was focused on the sample surface using a 20×/0.4 microscope magnification lens. Three 25-s accumulations were acquired to obtain all spectra.

Temperature-programmed reduction by hydrogen (H_2-TPR) was performed using an AutoChem II 2920 apparatus equipped with a thermal conductivity detector. Before measurement,

each sample was purified in situ for 1 h at 200 °C under He flow (heating rate: 10 °C/min; flow rate: 30 mL/min). Then, the sample was cooled to 25 °C, and TPR was performed using an H2(5%)/Ar mixture at 5 °C/min to 600 °C with a gas flow of 30 mL/min.

Temperature-programmed desorption (TPD-MS) was performed using an AutoChem II 2920 apparatus equipped with a thermal conductivity detector. Initially, 100 mg of solid catalyst was used. Before measurement, each sample was dried at 120 °C under an Ar flow (heating rate: 10 °C/min; flow rate: 15 mL/min) for 15 min. Then, the sample was cooled to 40 °C, and TPD measurements were performed using a He flow at a heating rate of 5 °C/min up to 800 °C and a flow rate of 15 mL/min. The MS data were collected using an OmniStar QMS-200 mass spectrometer (Pfeiffer Vacuum).

For elemental analysis, the 50 mg solid catalyst was digested with 10 mL concentrated HCl under microwave conditions (Ethos EASY - Rotor 44), followed by further dilution with water. The resulting solutions were analyzed using atomic absorption spectrometers (Thermo Fisher Scientific, Model iCE 3300).

Complementary results

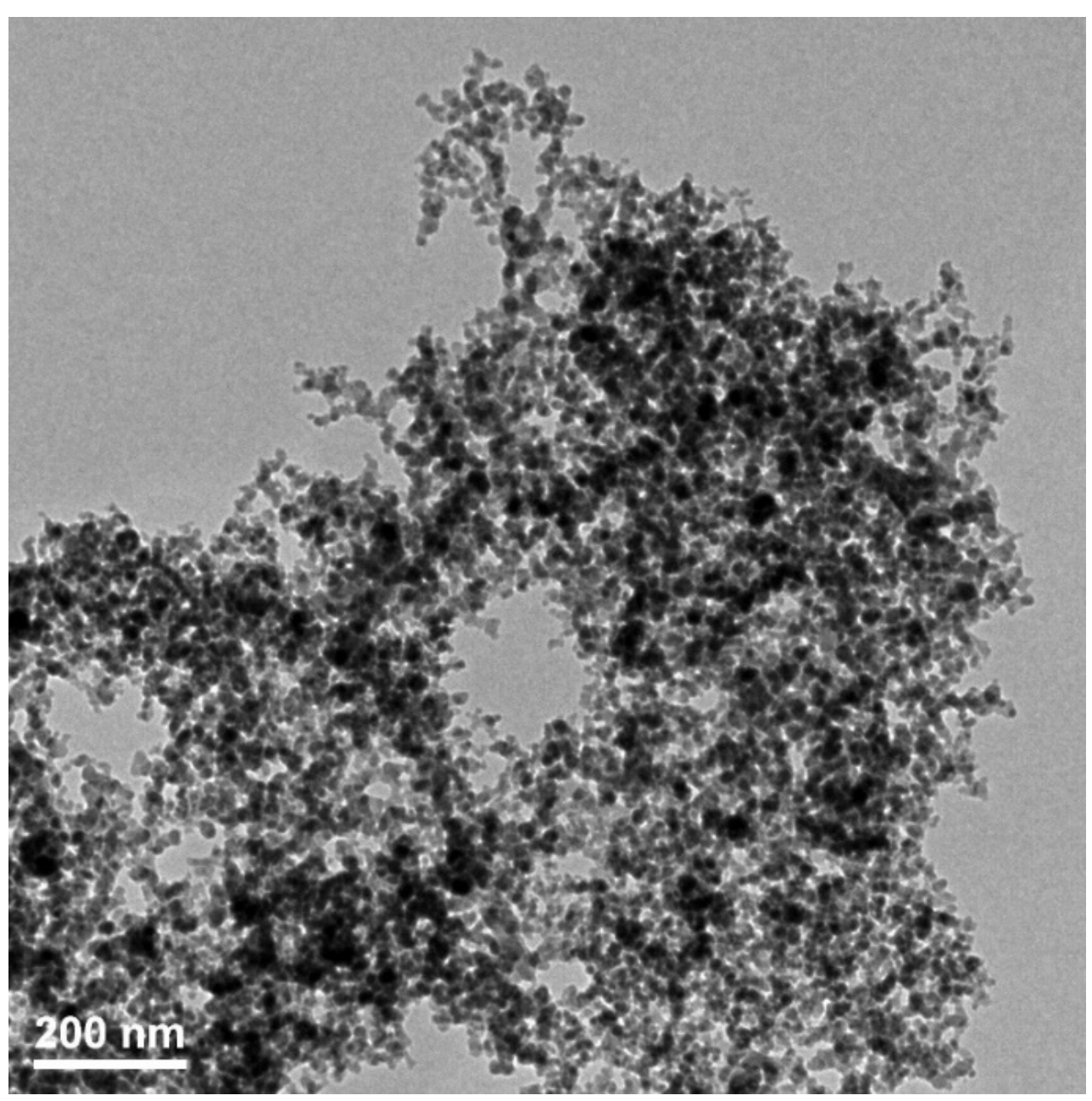

Figure S 1TEM image of Alu C 805.

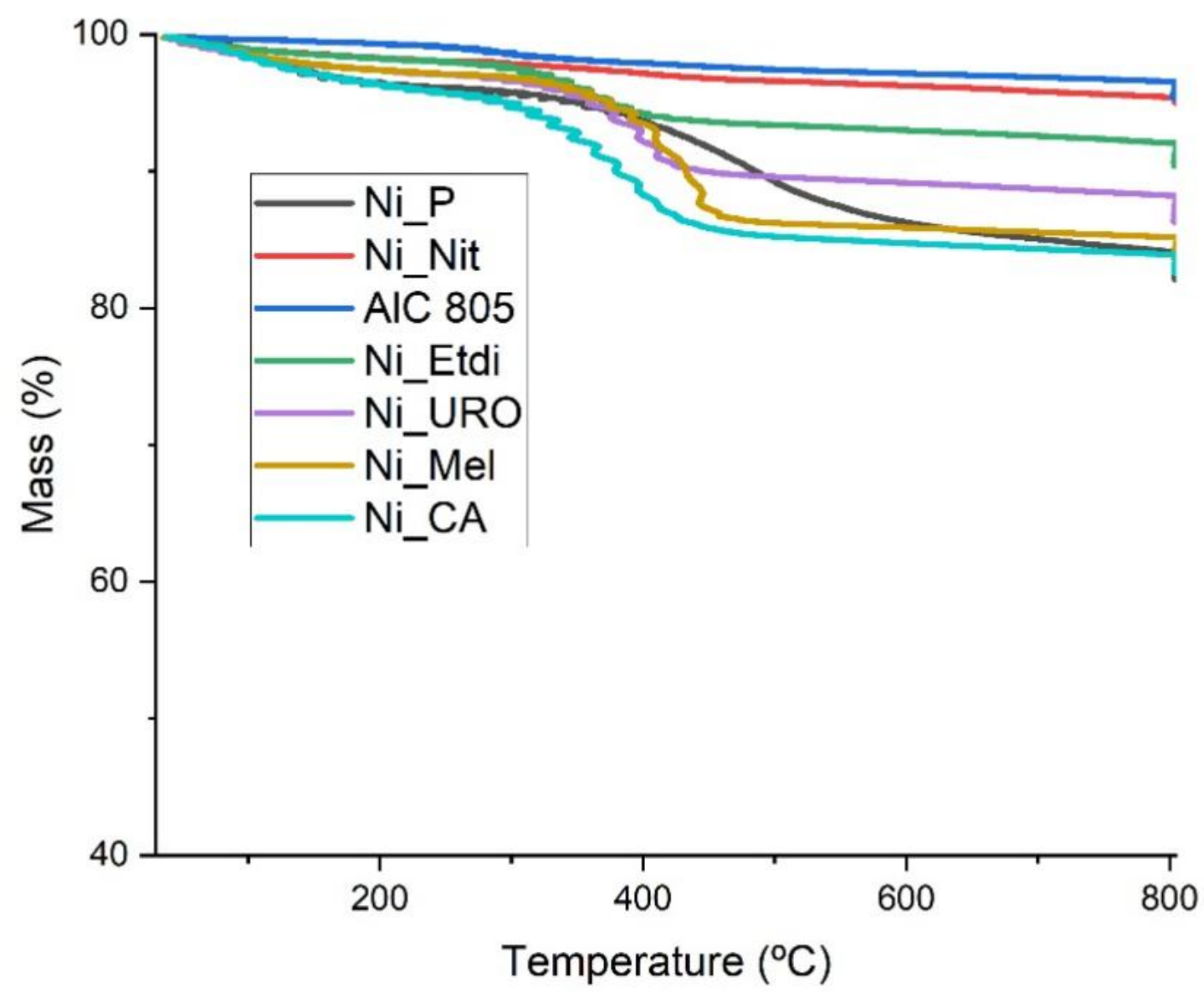

Figure S 2 TGA of Ni-based catalysts and Alu C 805

Table S 1 Mass loss of Ni-based catalyst and the pristine support (Alu C 805) obtained by TGA.

Material	Mass loss (%)
Alu C 805	4.7
Ni_Nit	5.0
Ni_Etdi	9.64
Ni_URO	13.72
Ni_Mel	16.33
Ni_CA	17.44
Ni_P	18

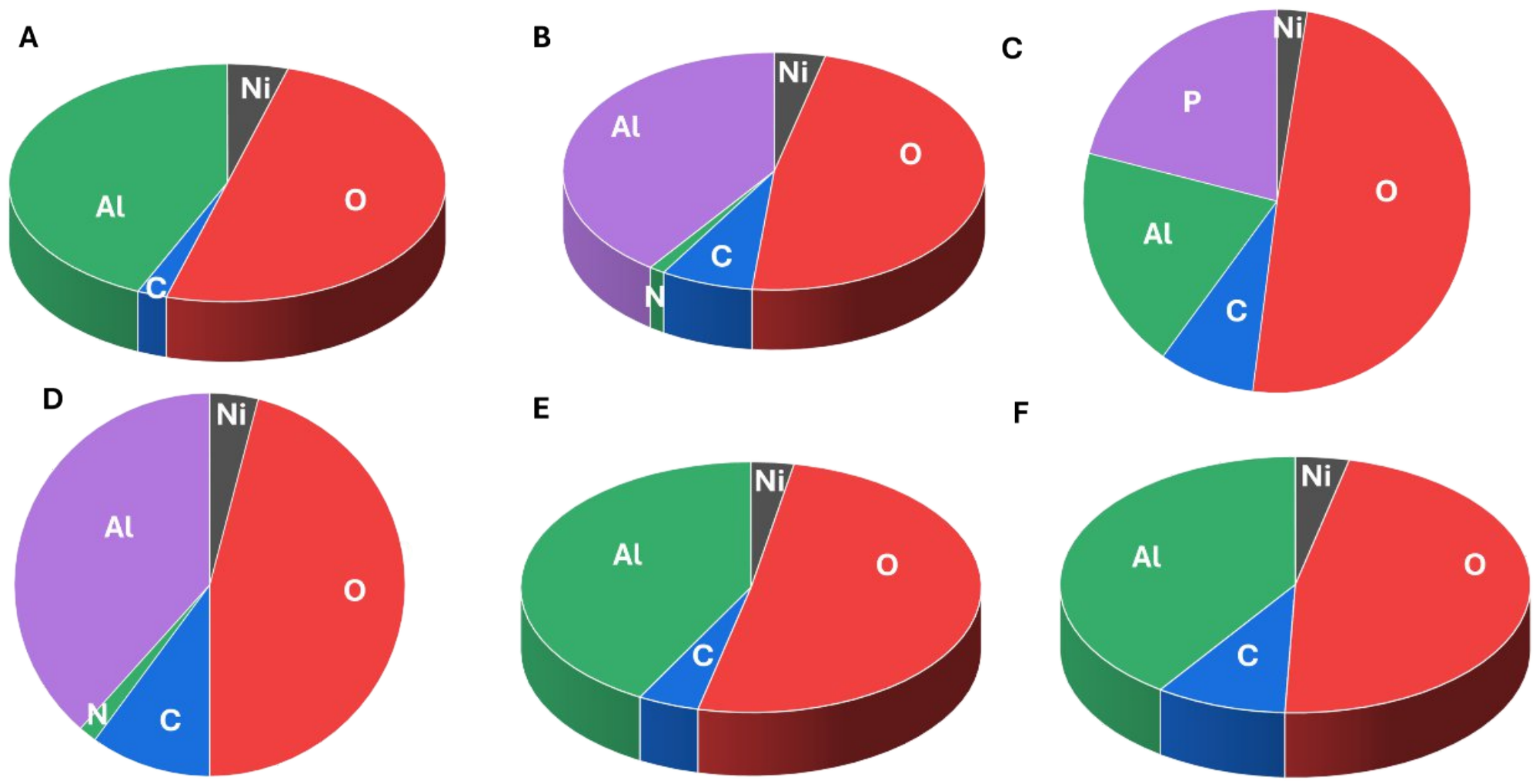

Figure S 3 Surface chemical composition of Ni-catalysts derived from XPS analyses (A) Ni_Nit; (B) Ni_URO; (C) Ni_P; (D) Ni_Mel; (E) Ni_Etdi; (F) Ni_CA.

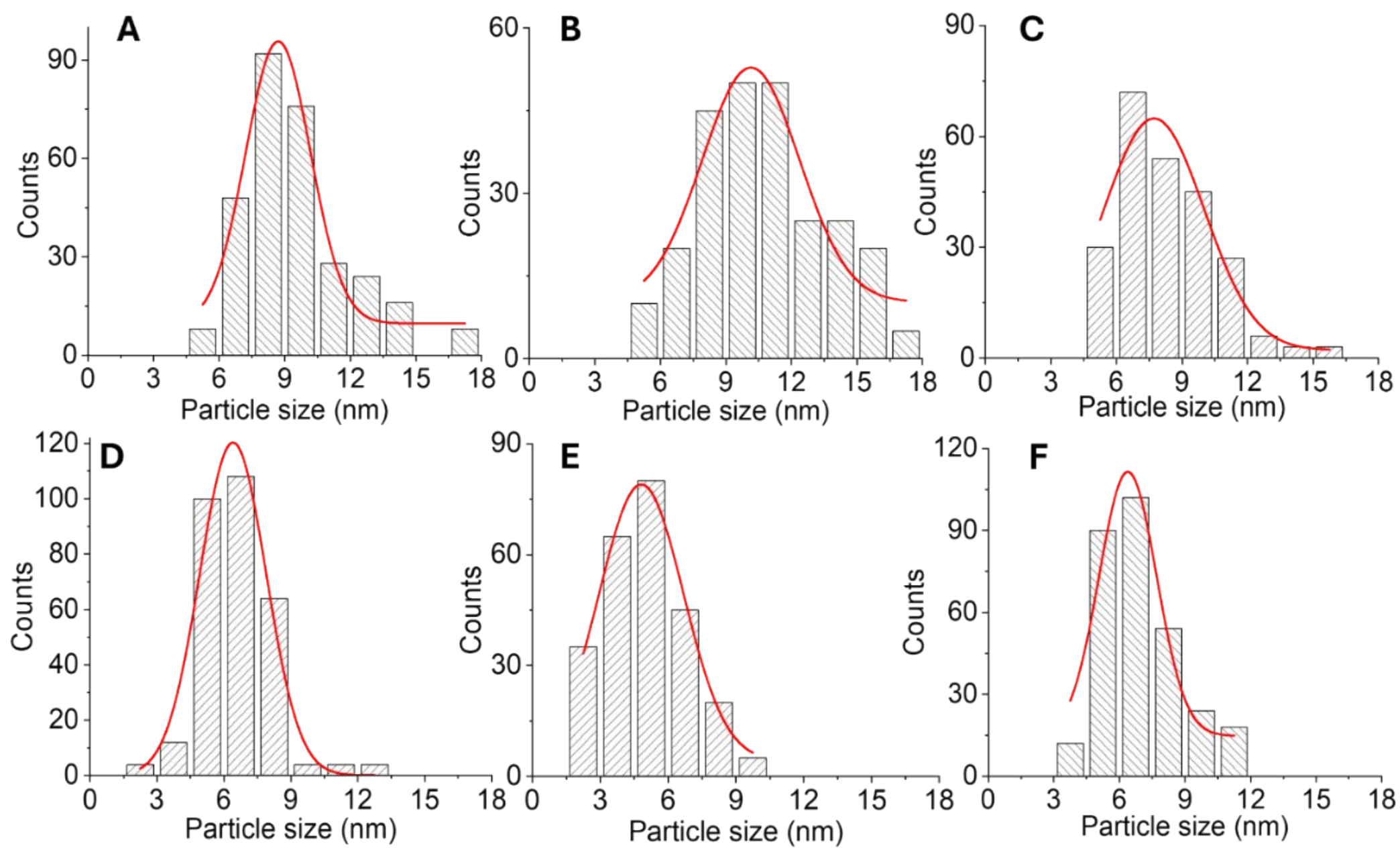

Figure S 4 Particle size distributions calculated using STEM HAADF data for: (A) Ni_Etdi; (B) Ni_Nit; (C) Ni_P; (D) Ni_URO; (E) Ni_CA; (F) Ni_Mel.

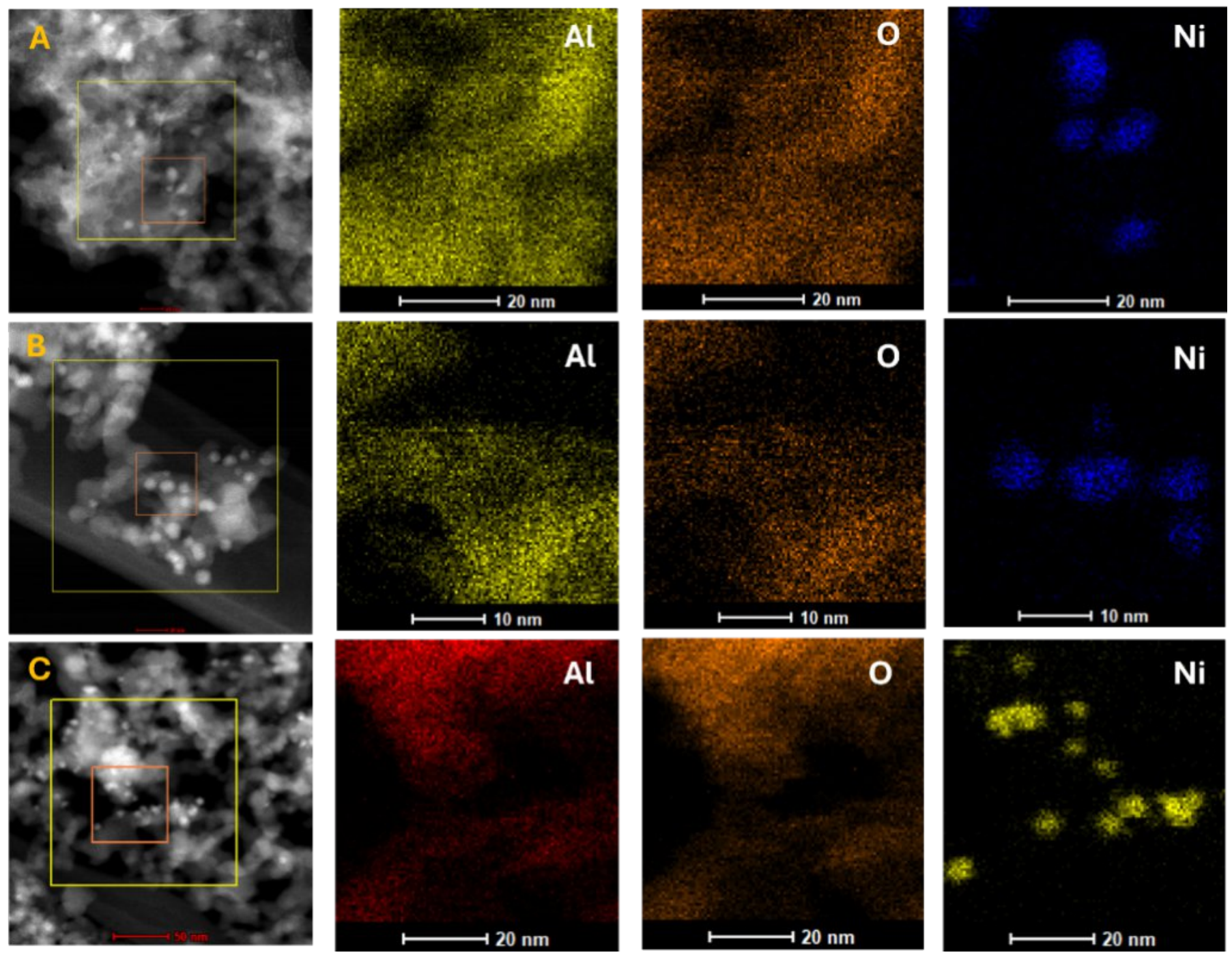

Figure S 5 EDS-STEM images of Ni_URO (A); Ni_Nit (B); and Ni_Mel (C) with the corresponding EDS maps of the orange area.

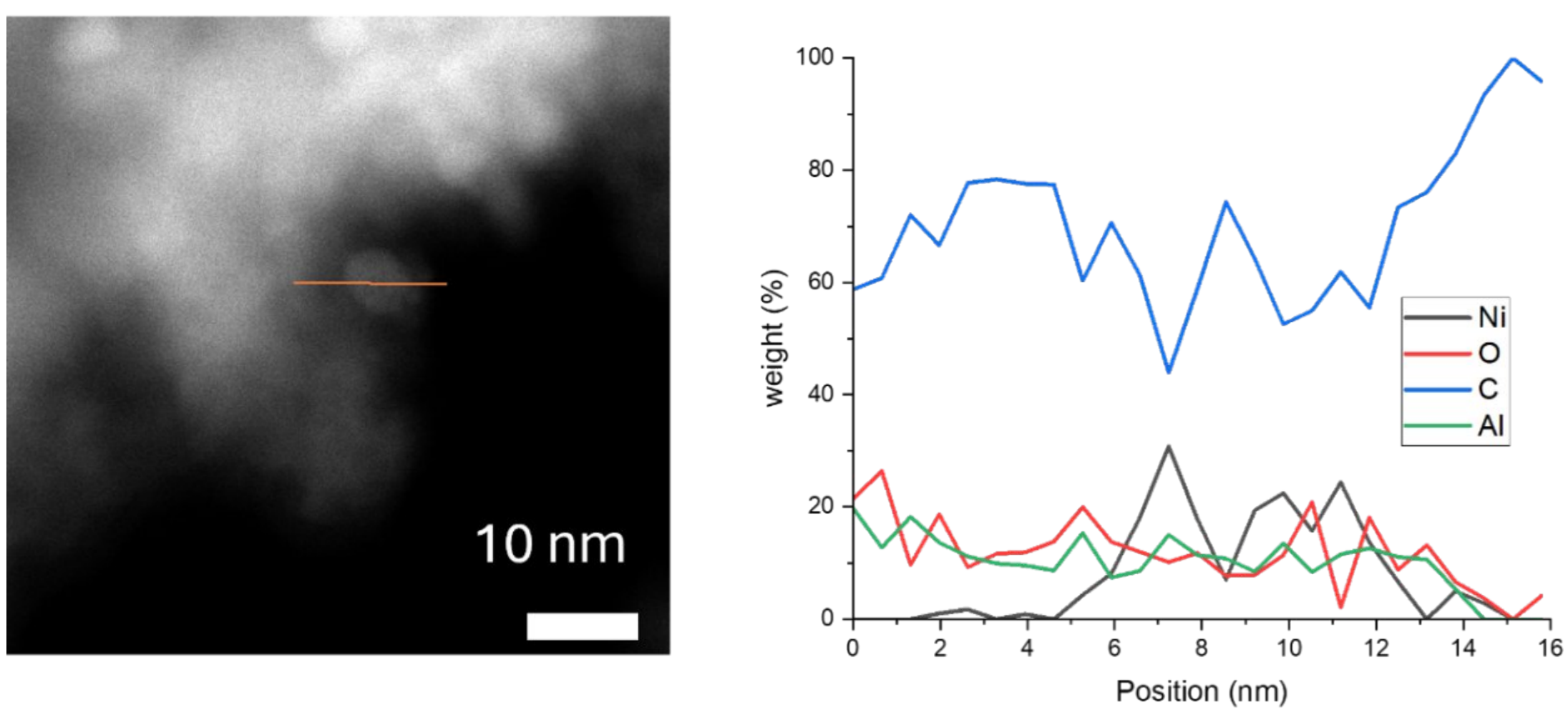

Figure S 6 HAADF-STEM image and its corresponding EDX line scan of Ni_CA.

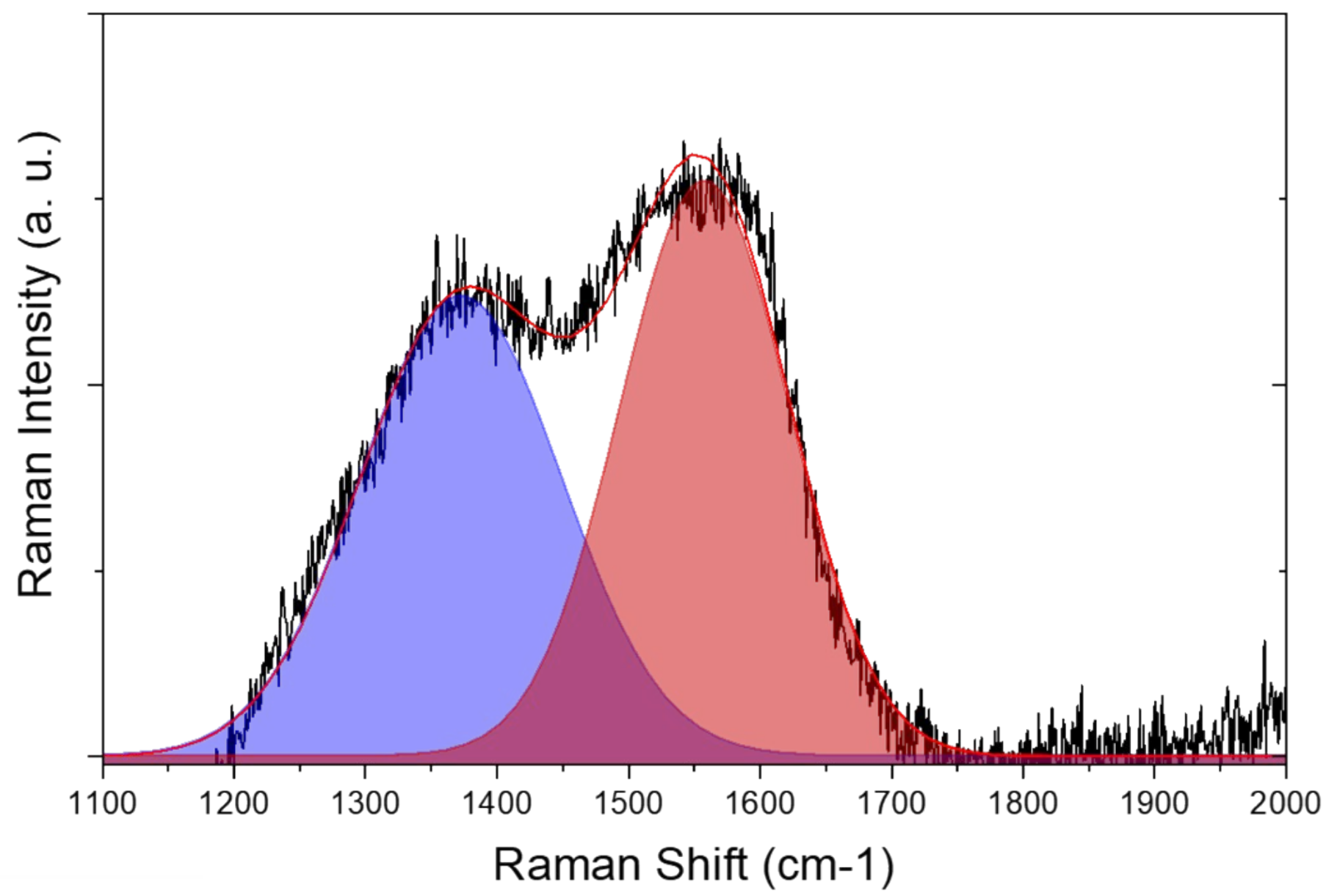

Figure S 7 Raman spectrum of Ni_CA.

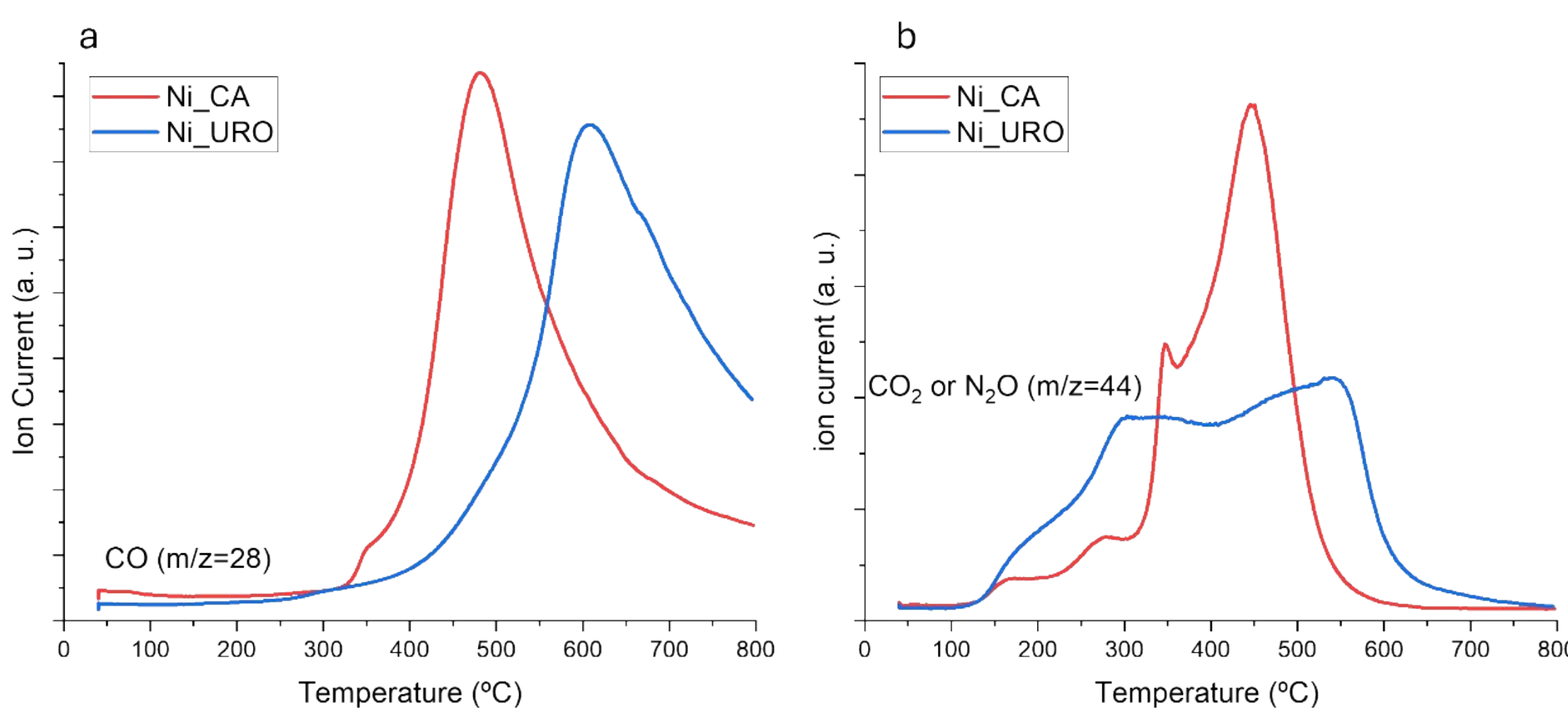

Figure S 8 Release of CO (a) and CO_2-N_2O (b) measured by TPD-MS.

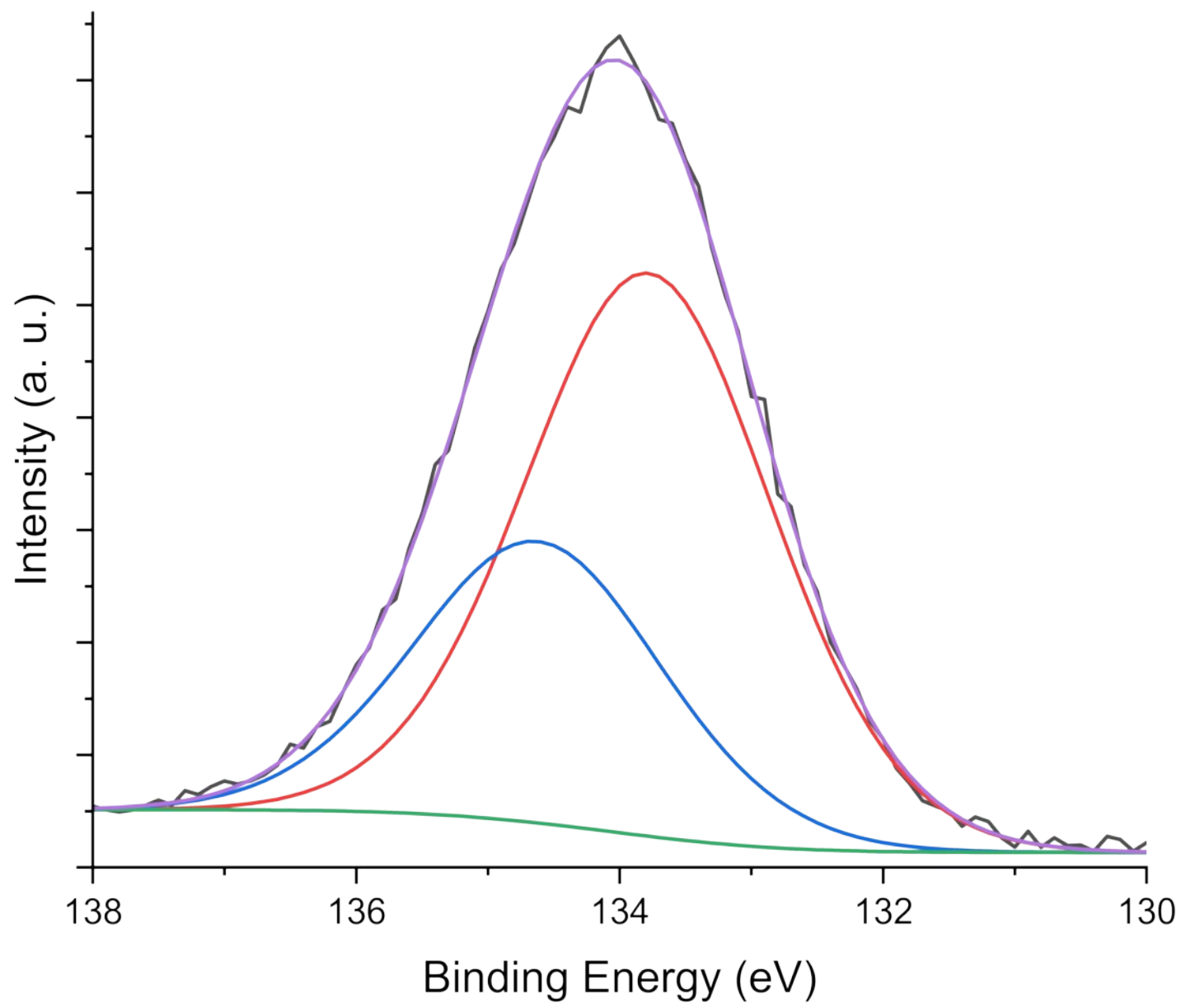

Figure S 9 High-resolution XPS spectrum of P 2p.

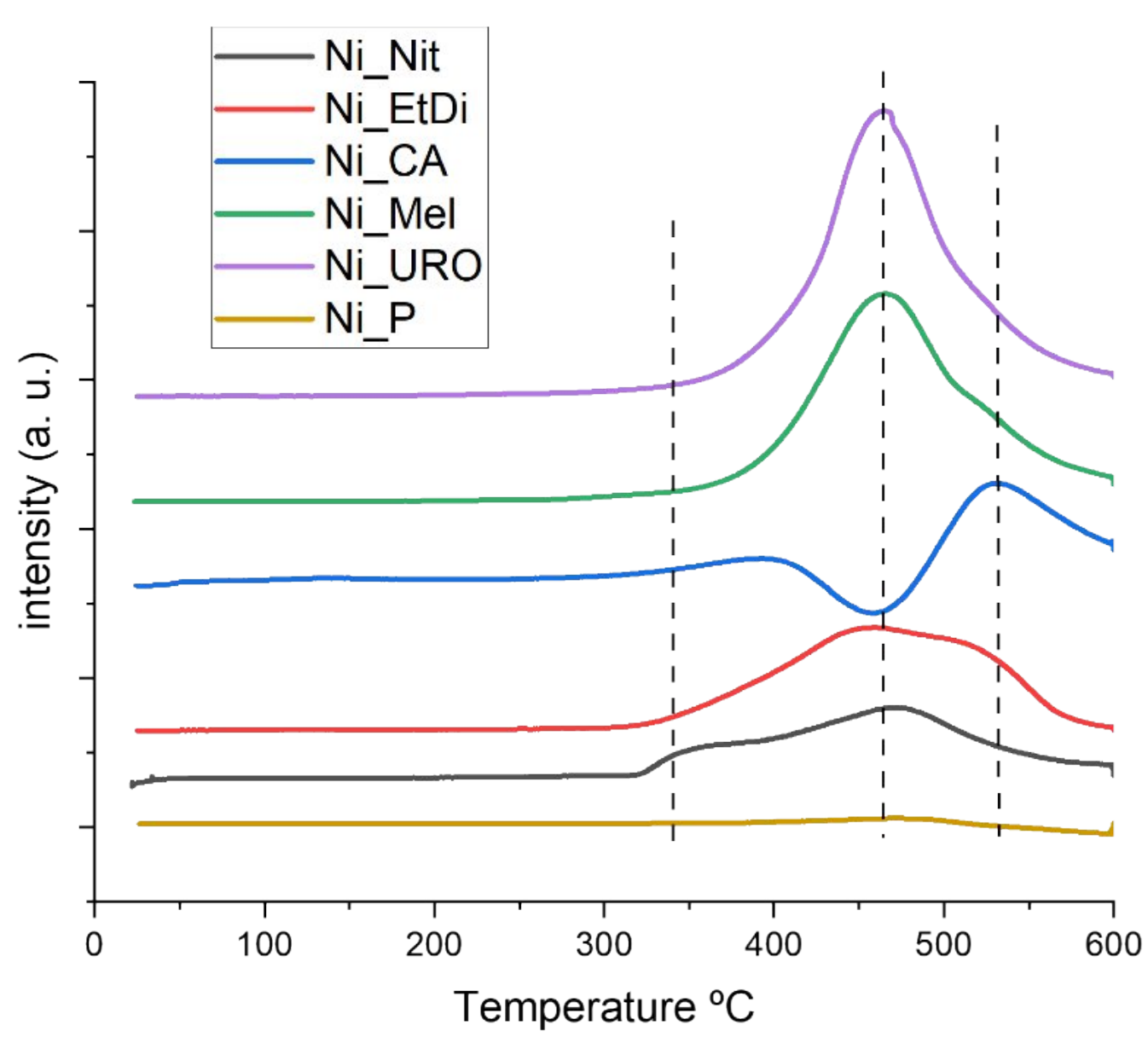

Figure S 10 TPR-H_2 of synthesized catalysts. The profiles were normalized by dividing each by the mass of the sample used for analysis.

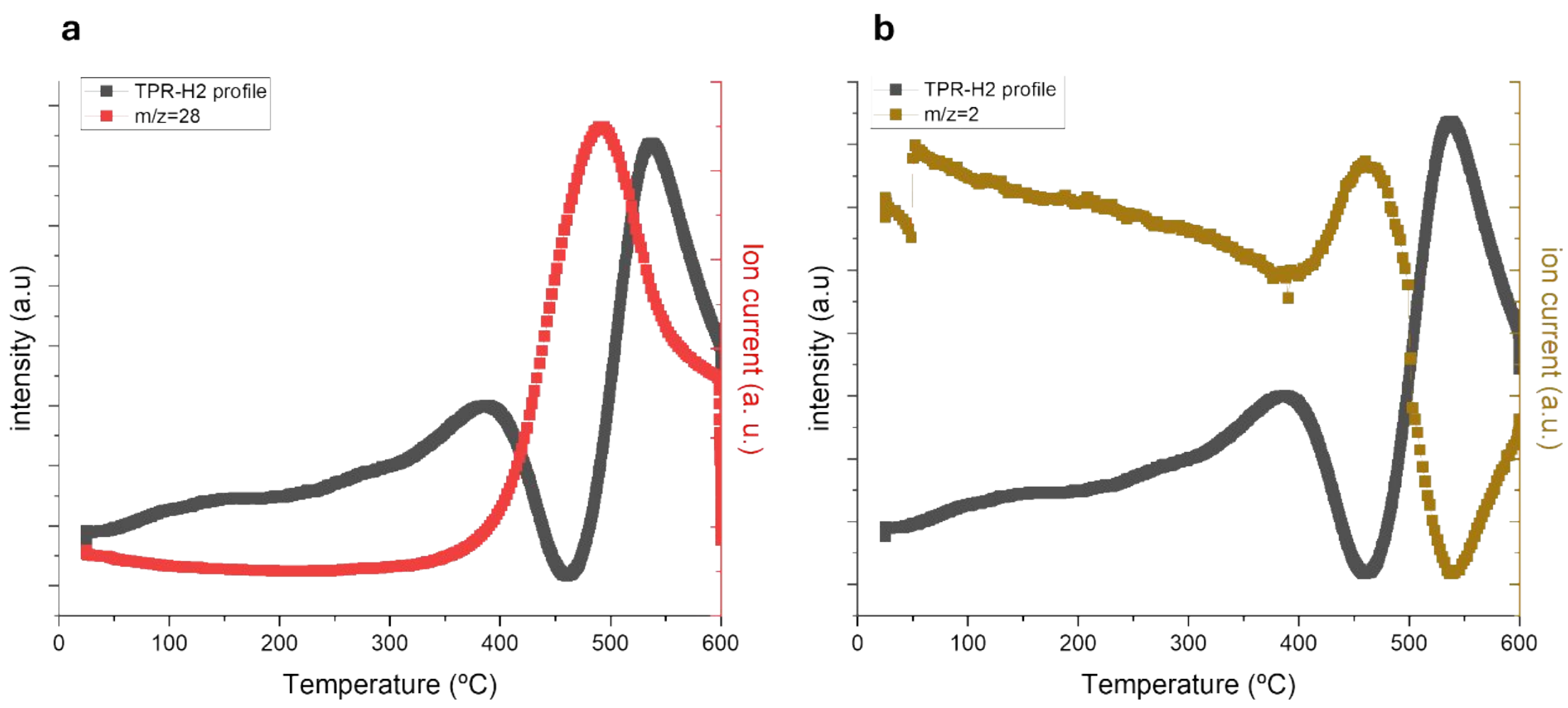

Figure S 11 TPR_H_2-MS correlated with (a) CO signal m/z=28; (b) hydrogen signal m/z=2.

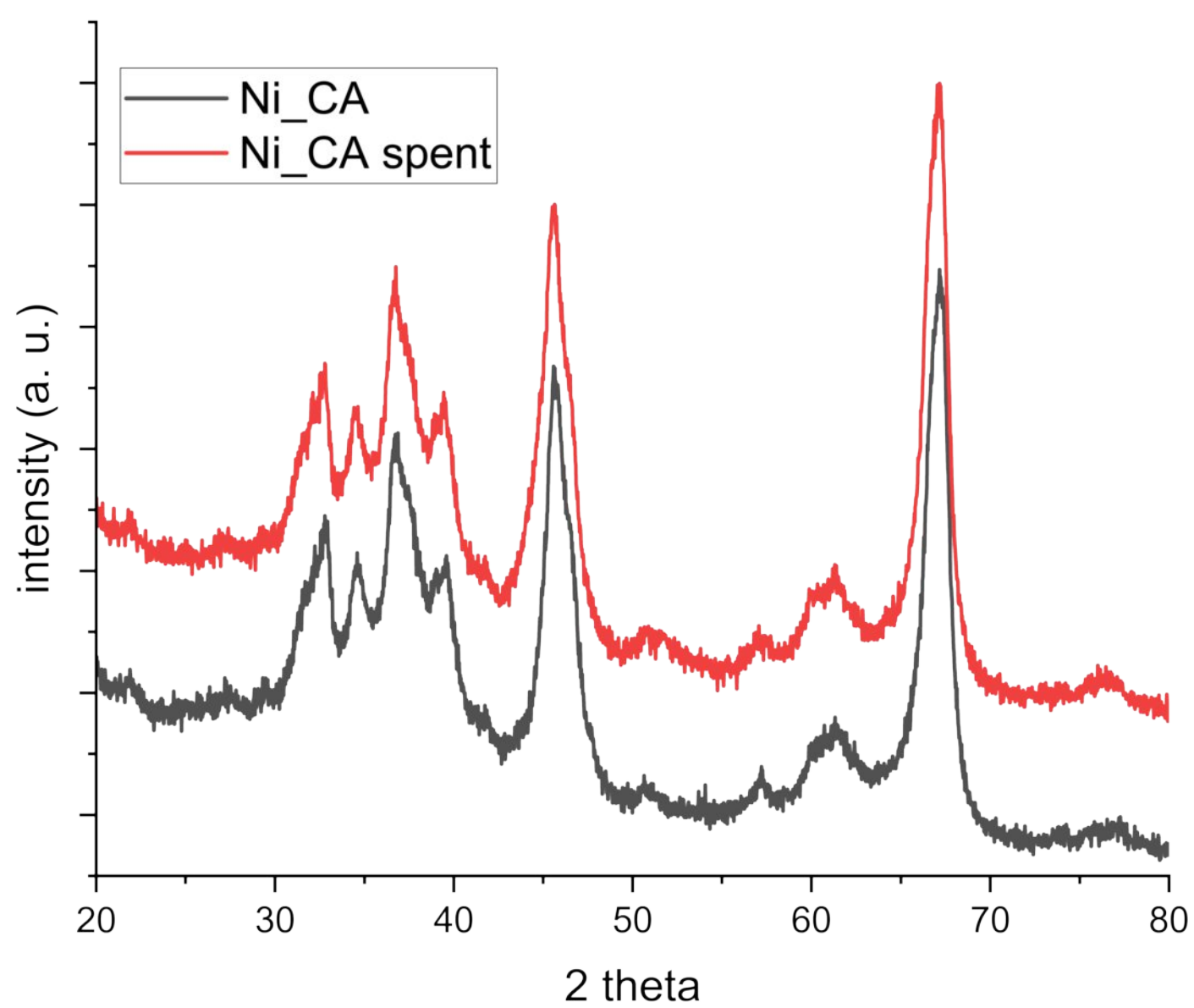

Figure S 12 XRD of fresh and spent Ni_CA catalyst.

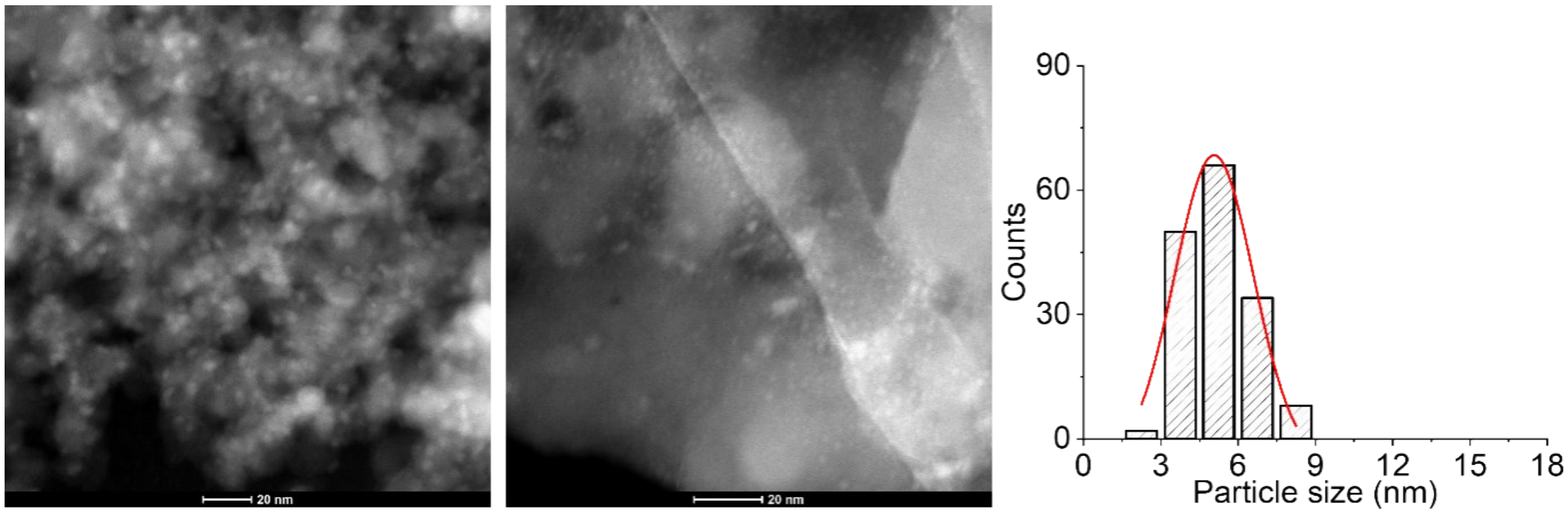

Figure S 13 HAADF-STEM images of spent Ni_CA after 3 runs conducted up to 550 °C, and the corresponding histogram of particle size distribution.

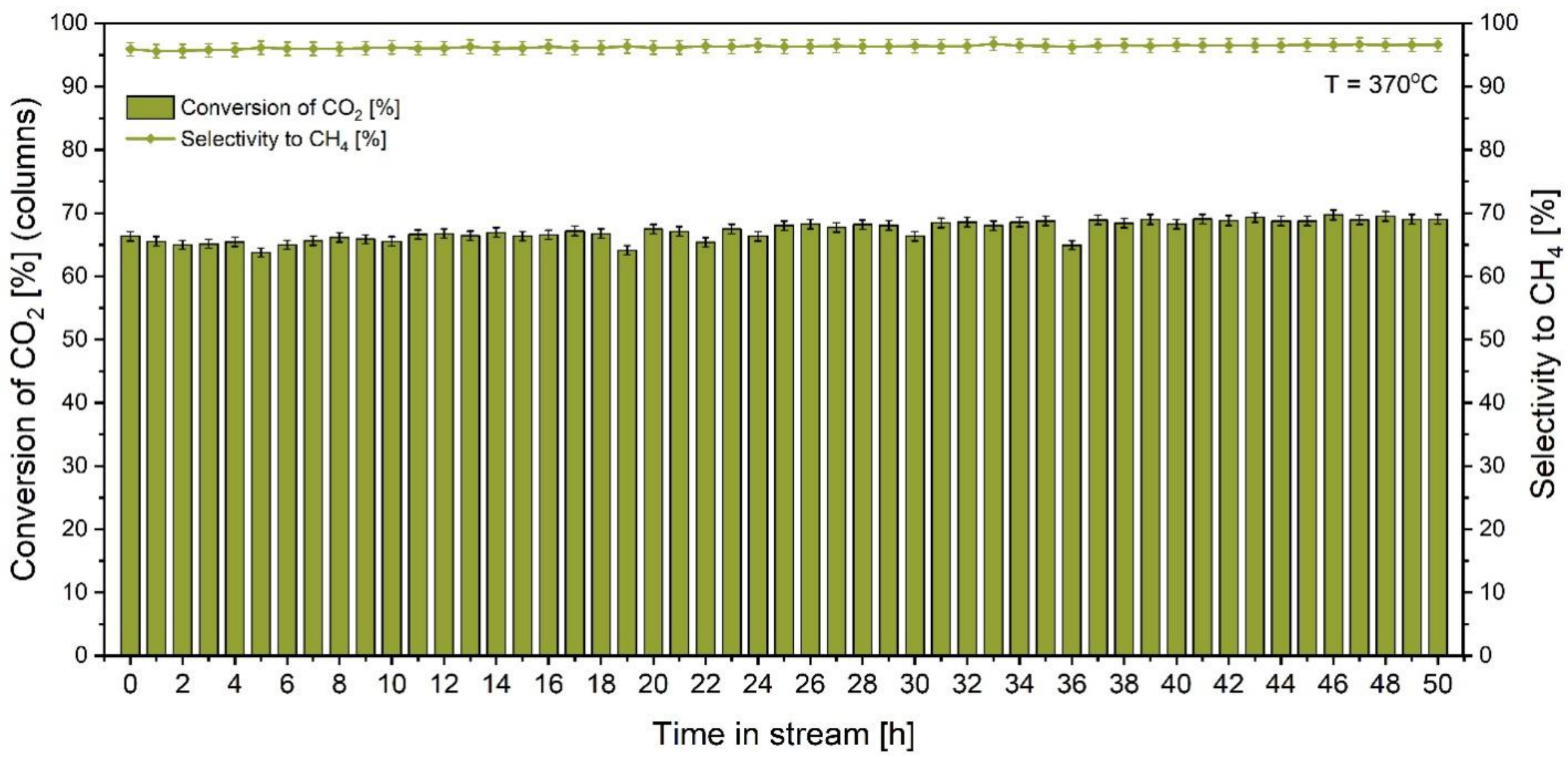

Figure S 14 Conversion and selectivity of CO_2 versus time (50 hours on stream).

Condition: 150 mg of catalyst (Ni_CA), 370ºC and $CO_2/H_2/He$ mixture at flow rates of 2, 8, and 40 mL·min⁻¹

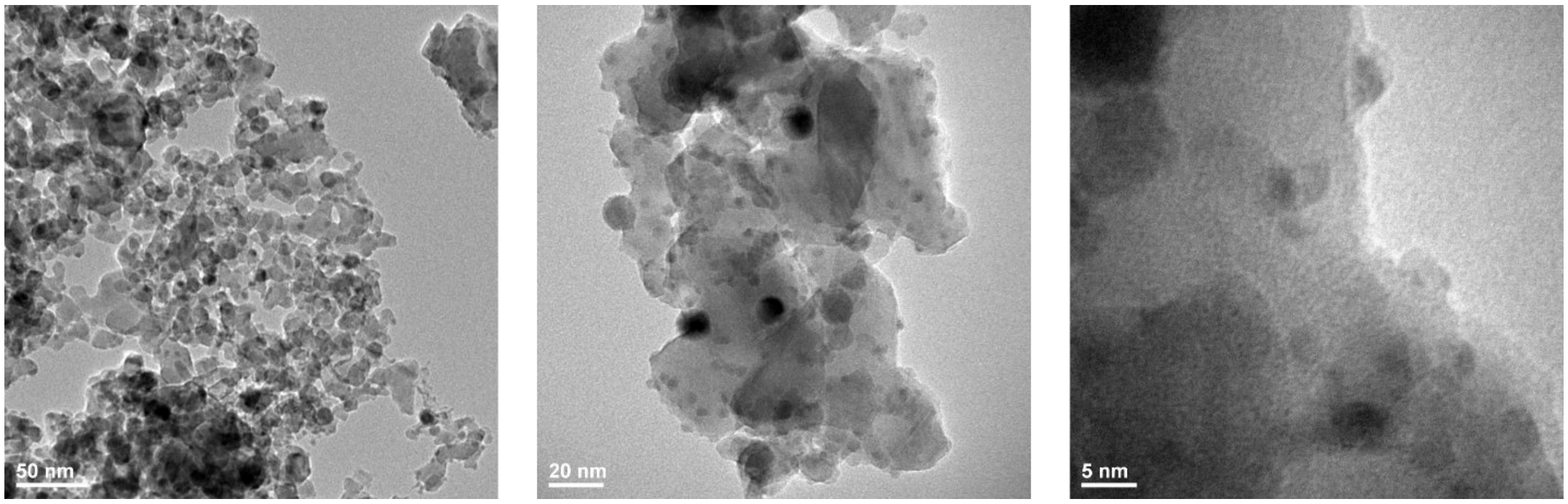

Figure S 15 TEM of spent catalyst after 50 hours on stream at 370ºC
